# Identification of Four New *agr* Quorum Sensing-Interfering Cyclodepsipeptides from a Marine *Photobacterium*

**DOI:** 10.3390/md11125051

**Published:** 2013-12-12

**Authors:** Louise Kjaerulff, Anita Nielsen, Maria Mansson, Lone Gram, Thomas O. Larsen, Hanne Ingmer, Charlotte H. Gotfredsen

**Affiliations:** 1 Department of Chemistry, Technical University of Denmark, DK-2800 Kgs. Lyngby, Denmark; E-Mail: lokja@kemi.dtu.dk; 2 Department of Veterinary Disease Biology, Faculty of Health and Medical Sciences, University of Copenhagen, DK-1870 Frederiksberg C, Denmark; E-Mail: anini@sund.ku.dk; 3 Department of Systems Biology, Technical University of Denmark, DK-2800 Kgs. Lyngby, Denmark; E-Mails: maj@bio.dtu.dk (M.M.); gram@bio.dtu.dk (L.G); tol@bio.dtu.dk (T.O.L.)

**Keywords:** *Photobacterium*, depsipeptide, structure elucidation, quorum sensing, antivirulence, *agr*

## Abstract

During our search for new natural products from the marine environment, we discovered a wide range of cyclic peptides from a marine *Photobacterium*, closely related to *P. halotolerans*. The chemical fingerprint of the bacterium showed primarily non-ribosomal peptide synthetase (NRPS)-like compounds, including the known pyrrothine antibiotic holomycin and a wide range of peptides, from diketopiperazines to cyclodepsipeptides of 500–900 Da. Purification of components from the pellet fraction led to the isolation and structure elucidation of four new cyclodepsipeptides, ngercheumicin F, G, H, and I. The ngercheumicins interfered with expression of virulence genes known to be controlled by the *agr* quorum sensing system of *Staphylococcus aureus*, although to a lesser extent than the previously described solonamides from the same strain of *Photobacterium*.

## 1. Introduction

The marine environment is a rich and vastly underexplored resource in many aspects. Most of the Earth’s surface is covered by water, inhabited by an incredible diversity of species, many of which have yet to be discovered. Microbial species are an important source for marine chemodiversity and it is believed that marine microorganisms will provide valuable drug candidates in the future [1,2]. There are however certain challenges with respect to sampling and culturing, and the usually low yields of metabolites can hamper full structural characterization and biological explorations.

A marine *Photobacterium* was selected from 500 bacterial strains collected during a global marine research cruise in 2006–2007 [3]. It was sampled from a mussel surface near the Solomon Islands and this bacterial strain was singled out as particularly interesting because of a two-pronged inhibitory effect of growth and quorum sensing (QS) in important human pathogens. The known pyrrothine antibiotic holomycin was responsible for the growth inhibition of *V. anguillarum* and *S. aureus* [4], while the solonamides were identified as the major contributors to the interference with *agr* quorum sensing in *S. aureus* [5]. Inhibition of virulence factor production and activity has been suggested as a new therapeutic approach suitable for antibiotic resistant pathogens [6]. In *S. aureus*, one of the possible targets is the *agr* quorum sensing system that in response to autoinducing peptide (AIP) accumulation at high cell densities induces expression of numerous extracellular toxins including *hla* encoding α-hemolysin while repressing expression of surface factors such as the *spa* encoded Protein A [7].

In this study, we report the identification of four cyclodepsipeptides in the 800–900 Da size range from the cell-associated (pellet) fraction of this marine *Photobacterium* that modulate expression of *agr* controlled virulence genes. The depsipeptides ngercheumicin F, G, H, and I are new additions to the structural family that initially constituted ngercheumicin A and B [8]. Three other depsipeptides ngercheumicin C, D, and E were isolated from the same bacterial strain as ngercheumicin A and B, but they are structurally different. All ngercheumicins reported to date have been isolated from *Photobacterium* spp. and a biological activity reported by Shizuri *et al.* for ngercheumicins A–E was against infections by *Pseudovibrio denitrificans* [8]. Like the solonamides [5], the ngercheumicins are 16-membered macrocyclic depsipeptides with some structural resemblance to the AIPs of *S. aureus*. Generally, AIPs consist of a cyclopentapeptide moiety cyclized through a cysteine residue by a thiolactonization, and with an exocyclic peptide chain of variable length extended from the cysteine residue in the *N*-terminal direction [9]. The exocyclic chain appears to be closely related to agonistic activity as truncated AIPs are known to have antagonistic properties [10]. Structure-activity relationship studies by Mayville *et al.* [11] indicated that adjacent Leu and Phe residues are required for inhibitory activity; however, structural comparisons of 24 natural staphylococcal AIPs later showed that they consistently have bulky, hydrophobic amino acid side chains in the *C*-terminus [12]. This may instead be the structural requirement for activity.

Here, we describe the isolation and structure elucidation of the four new ngercheumicins and discuss their role in QS.

## 2. Results and Discussion

### 2.1. Isolation and Structure Elucidation of Ngercheumicins F–I

The pellet of the *Photobacterium* sp. was extracted with organic solvents (see Experimental Section 3.2) and fractionated on a diol column. Mass spectrometric analysis revealed a series of peptide-like analogues which display good ionization in ESI^+^ MS and end absorption in UV spectroscopy. The fractions containing these analogues were pooled and subjected to further purification, first on a smaller diol column and then by preparative reversed phase HPLC, which gave four fractions of ngercheumicin F, G, H, and I, respectively. Ngercheumicins A and B were also detected by LC-MS, but they were not purified in sufficient amounts for structural or biological screening studies. The four new ngercheumicins were found to inhibit transcription of the regulatory *rnaIII* in *S. aureus*, which is the effector molecule of the *agr* QS system (See Section 2.2 and Supplementary Information).The ngercheumicins were isolated as white solids with the respective exact masses (HR-ESI-TOF) and molecular formulae: Ngercheumicin F (*m/z*: [M + H]^+^ 853.5685, calculated for C_43_H_77_N_6_O_11_ as 853.5650), ngercheumicin G (*m/z*: [M + H]^+^ 855.5907, calculated for C_43_H_79_N_6_O_11_ as 855.5807), ngercheumicin H (*m/z*: [M + H]^+^ 881.6033, calculated for C_45_H_81_N_6_O_11_ as 881.5963), and ngercheumicin I (*m/z*: [M + H]^+^ 883.6255, calculated for C_45_H_83_N_6_O_11_ as 883.6120).

**Figure 1:**
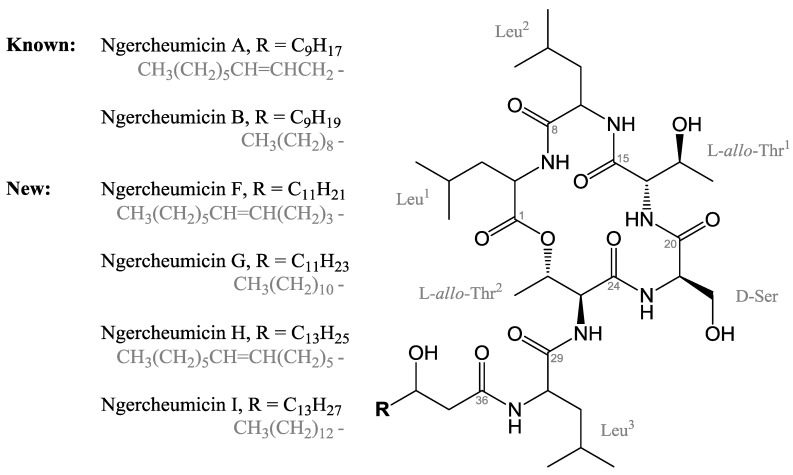
Structures of ngercheumicin A, B, F, G, H, and I, where ngercheumicin A, F, and H have an unsaturation (*cis*) in the unbranched alkyl chain, R.

Analysis of 1D and 2D nuclear magnetic resonance (NMR) spectroscopic data obtained for the four compounds characterized the structures as cyclodepsipeptides consisting of six amino acids and a 3-hydroxy fatty acid (six NH signals and seven carbonyl resonances) (Figure 1). All four ngercheumicins were elucidated as having identical amino acid sequences, consisting of three leucines, two threonines, and one serine, as established by DQF-COSY, gHSQC, gHMBC, gH2BC, and TOCSY 2D NMR spectroscopic analyses. The closure of the macrocyclic ring through an ester linkage between the *C*-terminus and the hydroxyl group in one of the threonine residues was verified by the low field chemical shift of the β-proton of Thr^2^ (H26, Table 1) and a HMBC correlation between H26 and the carbonyl (C1) of the *C*-terminal Leu^1^ residue (for HMBC and H2BC correlations, see Supplementary Information). This formed a 16-membered macrocycle with an exocyclic chain continuing in the *N*-terminal direction from Thr^2^, the chain constituting a Leu residue and a 3-hydroxy fatty acid (Figure 1). The structural difference between the four analogues was found in the length and saturation of the unbranched fatty acid chain. Ngercheumicin F and H each have one double bond in the 3-hydroxy fatty acid chain, whereas the fatty acids in ngercheumicin G and I are fully saturated, but with the same lengths as F and H, respectively. The previously isolated ngercheumicin A and B have a similar relationship, but with 12-carbon long fatty acid chains [8], whereas ngercheumicin F and H have fatty acid chains with 14 and 16 carbon atoms, respectively. The longer chains result in increasing overlap of ^1^H and ^13^C resonances in the aliphatic regions on both sides of the double bond. By thorough examination of HMBC and H2BC correlations in the chains, the structures of ngercheumicin F and H were elucidated and assigned as shown in Figure 1 and Table 1, respectively. The chemical shifts of ngercheumicin H were virtually symmetrical around the double bond because of the long chain, whereas there was a slight shift in ngercheumicin F, which has a shorter alkyl chain. The position of the double bond in ngercheumicin H was tentatively assigned based on the NMR data, as the data left an ambiguity of a CH_2_-group between position 41 and 47 due to the high degree of symmetry and the overlapping resonances that 2D NMR was unable to resolve. However, assuming a correct assignment, a structural pattern emerged where ngercheumicin A, F, and H had their double bonds positioned seven carbon atoms from the end of the fatty acid chain, also known as *n*-7 fatty acids. Counting from the peptide-end of the fatty acid chain, the double bonds in ngercheumicin F and H are thus situated further into the fatty acid chains.

**Table 1:** ^1^H and ^13^C NMR spectroscopic data (800 MHz, DMSO-*d*_6_) for ngercheumicins F–I. More elaborate NMR tables can be found in the Supplementary Information.

	Ngercheumicin F		Ngercheumicin G		Ngercheumicin H		Ngercheumicin I	
Position, Type	δ_H_ (ppm)	δ_C_ (ppm)	δ_H_ (ppm)	δ_C_ (ppm)	δ_H_ (ppm)	δ_C_ (ppm)	δ_H_ (ppm)	δ_C_ (ppm)
*Leu^1^*								
1—CO	−	170.8	−	170.8	−	170.8	−	170.8
2—CH_α_	4.37	51.1	4.37	51.1	4.37	51.1	4.37	51.1
3—CH_β_	1.62, 1.55	40.4	1.61, 1.55	40.4	1.62, 1.56	40.4	1.61, 1.56	40.4
4—CH_γ_	1.49	24.5	1.49	24.5	1.50	24.5	1.49	24.5
5—CH_δ,1_	0.88	22.3	0.88	22.3	0.89	22.3	0.88	22.3
6—CH_δ,2_	0.85	22.5	0.85	22.5	0.85	22.5	0.85	22.5
7—NH	7.73	−	7.73	−	7.73	−	7.73	−
*Leu^2^*								
8—CO	−	171.0	−	171.0	−	171.0	−	171.0
9—CH_α_	4.26	50.4	4.26	50.4	4.25	50.4	4.26	50.4
10—CH_β_	1.49	38.8	1.49	38.8	1.49	38.8	1.49	38.8
11—CH_γ_	1.53	24.2	1.53	24.2	1.54	24.1	1.54	24.1
12—CH_δ,1_	0.87	22.9	0.87	22.9	0.87	22.9	0.87	22.9
13—CH_δ,2_	0.80	21.4	0.80	21.4	0.80	21.4	0.80	21.4
14—NH	8.12	−	8.11	−	8.11	−	8.11	−
l*-allo-Thr^1^*								
15—CO	−	170.7	−	170.7	−	170.7	−	170.7
16—CH_α_	3.86	59.9	3.86	59.9	3.86	59.9	3.86	59.9
17—CH_β_	3.88	65.1	3.88	65.1	3.88	65.1	3.87	65.1
17—OH	4.70	−	4.69	−	4.69	−	4.69	−
18—CH_γ_	1.03	20.0	1.03	20.1	1.03	20.1	1.03	20.1
19—NH	8.09	−	8.09	−	8.09	−	8.09	−
d*-Ser*								
20—CO	−	170.0	−	170.0	−	170.0	−	170.0
21—CH_α_	4.38	54.5	4.38	54.6	4.38	54.5	4.38	54.5
22—CH_β_	3.54	61.6	3.54	61.5	3.54	61.5	3.54	61.5
22—OH	4.86	−	4.86	−	4.86	−	4.86	−
23—NH	7.53	−	7.53	−	7.53	−	7.53	−
l*-allo-Thr^2^*								
24—CO	−	168.4	−	168.4	−	168.4	−	168.4
25—CH_α_	4.40	56.2	4.39	56.2	4.39	56.2	4.39	56.2
26—CH_β_	5.35	69.6	5.34	69.6	5.34	69.6	5.35	69.6
27—CH_γ_	1.10	17.0	1.10	17.0	1.10	16.9	1.10	17.0
28—NH	8.40		8.39		8.39		8.39	
*Leu^3^*								
29—CO	−	173.1	−	173.2	−	173.1	−	173.2
30—CH_α_	4.37	51.7	4.37	51.8	4.37	51.8	4.37	51.8
31—CH_β_	1.48	40.3	1.48	40.2	1.48	40.2	1.48	40.2
32—CH_γ_	1.62	24.2	1.62	24.2	1.62	24.2	1.63	24.2
33—CH_δ,1_	0.92	22.6	0.92	22.6	0.91	22.6	0.92	22.6
34—CH_δ,2_	0.87	21.9	0.87	21.9	0.87	21.9	0.87	21.9
35—NH	8.20	−	8.20	−	8.20	−	8.20	−
*Fatty acid*								
36—CO	−	171.8	−	171.9	−	171.9	−	171.9
37	~2.24	43.3	2.24, 2.22	43.4	2.25, 2.22	43.4	2.25, 2.22	43.4
38	3.78	67.4	3.77	67.5	3.78	67.5	3.78	67.5
38—OH	4.62	−	4.60	−	4.60	−	4.59	−
39	1.34, 1.30	36.2	1.31	36.6	1.32	36.6	1.32	36.6
40	1.42, 1.28	25.1	1.34, 1.22	24.8	1.36, 1.23	24.8	1.35, 1.22	24.8
41	1.96	26.6	1.23	~29	~1.2	~29	1.23	29.1
42	5.31	129.6	~1.2	~29	1.28	~29	~1.2	~29
43	5.31	129.6	~1.2	~29	1.97	26.6	~1.2	~29
44	1.96	26.5	~1.2	~29	5.31	129.6	~1.2	~29
45	1.28	29.0	~1.2	~29	5.31	129.6	~1.2	~29
46	1.24	28.3	1.21	29.1	1.97	26.6	~1.2	~29
47	1.22	31.1	1.22	31.3	1.28	~29	~1.2	~29
48	1.25	22.1	1.25	22.1	1.25	28.2	~1.2	~29
49	0.84	13.9	0.84	13.9	1.22	31.1	1.22	31.3
50					1.25	22.0	1.25	22.1
51					0.84	13.9	0.84	13.9

The resonances originating from the double bond were very close in chemical shift, leading to severe second order effects in the ^1^H multiplet patterns. Due to the second order spin systems, it was not possible to determine the size of the *J* coupling constant, however the total span of the multiplets at 5.31 ppm for both F and H was below 15 Hz, and assuming complete symmetry, a *trans* coupling seemed unlikely. The chemical shifts for the allylic carbons at 26.5–26.6 ppm are also consistent with *cis* configuration, as allylic carbons in *trans* fatty acids are about 5 ppm further downfield [13]. Therefore the double bonds in ngercheumicin F and H were assigned as *cis*.

Attempts to obtain absolute stereochemical assignment of the amino acids were done by Marfey’s method, using existing methods for acid hydrolysis [14] and derivatization with Marfey’s reagent [15]. Complete stereochemical assignments were not obtained as Marfey’s method revealed the presence of both l- and d-Leu, and unambiguous resolution of the hydroxyamino acids Ser and Thr is known to be challenging [16]. Pure enantiomers of Leu, Ser and Thr were used to synthesise single diastereomers with Marfey’s reagent for comparison with the Marfey’s derivatives of the hydrolysed ngercheumicins. This also included *allo*-Thr. By comparison to the pure amino acid derivatives, l-Ser, d-Thr and d-allo-Thr were dismissed. Therefore the configuration of the Ser residue was firmly assigned as d-Ser. Although the peaks of l-Thr, d-Ser, and l-*allo*-Thr eluted within a narrow spectral window, the elution order together with MS detection verified the presence of l-*allo*-Thr and no l-Thr. This was supported by the size of the *J* coupling constant from Thr^2^ H26 to H25 and H27. The absolute configuration of the three Leu residues was ambiguous, however both l- and d-Leu were present. Due to the minute amounts available, (0.5–1.1 mg of each analogue) the configuration of the 3-hydroxy fatty acid was not determined.

Organic synthesis could be a solution to the supply problem of these peptides, as synthesis of cyclic peptides is often a relatively straightforward procedure. Many cyclic peptides from marine microbial sources contain non-proteinogenic and d-amino acids as well as polyketide-derived structural motifs or fatty acids [17], which is also the case for the solonamides and the ngercheumicins. Undoubtedly, these traits complicate synthesis of both natural products and analogues. However, a study by Molhoek *et al.* showed that cyclization of a peptide antibiotic and substitution of l- with d-amino acids improved stability towards bacterial proteases (including those in *S. aureus*) and decreased cytotoxicity while retaining the antibacterial activity [18]. The ngercheumicin macrocycle is closed through an ester bond between the *C*-terminus and the side chain of a Thr residue. This is a common trait in depsipeptides, and e.g., the cyclodepsipeptide plitidepsin [2], which is undergoing clinical trials for treatment of several cancers, has this feature in common with the ngercheumicins.

### 2.2. Ngercheumicins Interfere with *agr*

Ngercheumicin F, G, H, and I were examined in the *S. aureus lacZ* reporter assays described by Nielsen *et al.* [19] monitoring transcriptional activity of the *hla*, *spa*, and P3 (*rnaIII*) promotors. All four ngercheumicins increased transcription of *spa* and reduced expression of *hla* and *rnaIII*, compared to a solvent control (see Supplementary Information). The inverse effect of the ngercheumicins on *spa* expression compared to *rnaIII* and *hla* indicates that the ngercheumicins interfere with *agr* activation.

**Figure 2:**
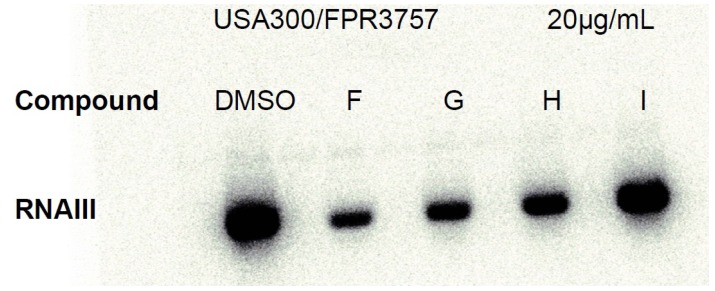
Northern blot of ngercheumicin F, G, H and I treated *S. aureus* USA300 wt (FPR3757) cells in stationary phase (OD_600_ = 3) at ngercheumicin concentration of 20 µg mL^−1^.

To confirm these initial results, cultures of a highly virulent, community-acquired strain of *S. aureus* USA300 were exposed to ngercheumicins F–I and RNAIII expression was monitored in exponential and stationary growth phase by Northern blot analysis. Data confirmed that ngercheumicins reduced expression of *rnaIII* and thus interfere with *agr* in CA-MRSA strain USA300. At the ngercheumicin concentrations used in the culture samples for Northern blotting, there was a minor down regulation of *rnaIII* by ngercheumicin G and H at 5 µg mL^−1^ (see Supplementary Information). At 20 µg mL^−1^ (23 μM, see Figure 2), the *rnaIII* inhibiting effect was more pronounced for ngercheumicin G and H, but also F showed activity here and appeared to have the highest *rnaIII* inhibiting activity. Ngercheumicin I did not show any significant effect at either 5 or 20 µg mL^−1^.

**Figure 3:**
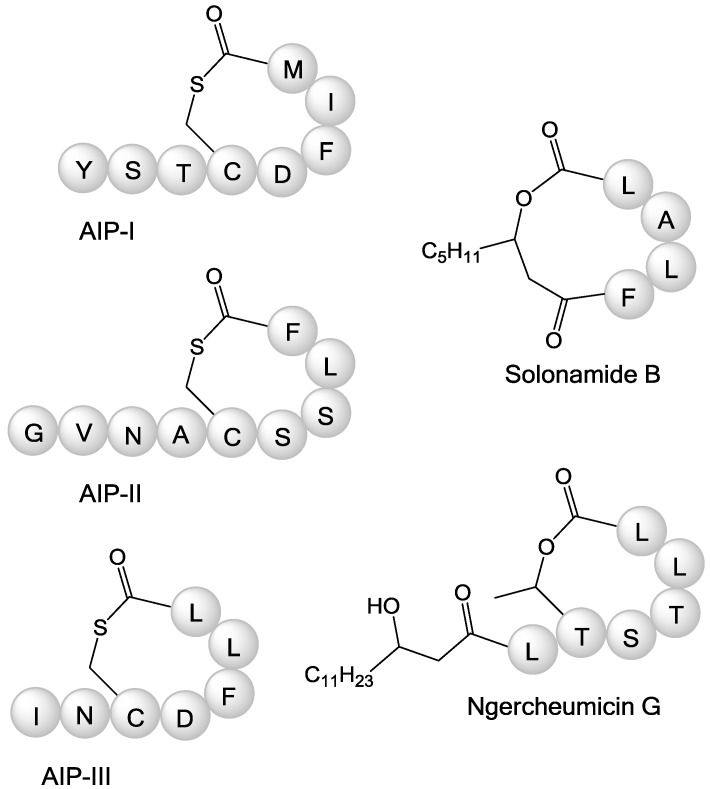
Schematic structures of *S. aureus* autoinducing peptides (AIPs) I–III, solonamide B and ngercheumicin G, with amino acid sequences and type of ring closure. AIP structures are reproduced from [21]. AIP-IV also exists, where the aspartic acid (D) of AIP-I is replaced by a tyrosine residue.

The ngercheumicins share structural traits with the AIPs of *S. aureus*. *S. aureus* strains express one of a least four variants of AIPs and each variant induces *agr* expression in strains of the same type but repress *agr* expression in strains of other types [20]. Interestingly, the ngercheumicins resemble the type II and III AIPs, whereas the solonamides resemble type I and II the most [21]. All have 16-membered macrocyclic rings and flexible exocyclic chains (Figure 3). The AIPs have purely peptidic exocyclic chains, whereas the depsipeptides all contain fatty acid chains, but both the AIPs and the cyclodepsipeptides have variations in the length of the exocyclic chains, which perhaps relate to receptor specificity. Looking at the amino acid sequence of the ngercheumicins (Figure 3) starting at the *C*-terminus, the two Leu residues are also found in AIP-III, and the Thr-Ser sequence closely resembles the Ser-Ser sequence in AIP-II. It should be noted that this comparison is made without taking 3D configuration of the side chains into account, because only the 2D structures are known. It is however clear that part of the backbone structure is highly homologous to the native AIPs of *S. aureus*.

Little is known about peptide signaling in the marine environment and whether other groups of peptide signal molecules exist there. It can be speculated that these depsipeptides interfere with QS pathways present in the marine environment or even act as alternative quorum sensing molecules. Existing QS systems include the *N*-acyl homoserine lactones in Gram-negative bacteria and the auto inducing peptides in the Gram-positive staphylococci [22]. That ngercheumicins F–I were isolated in low yields as intracellular metabolites, unlike the solonamides that were excreted in large amounts, could mean that they do not act as intercellular signaling molecules in the natural environment and therefore there could be an unexplored biological role for the ngercheumicins. Analyses of co-cultures of this *Photobacterium* with naturally co-occurring or competitive strains could provide further insight into the biological function of these compounds.

## 3. Experimental Section

### 3.1. Isolation and Identification of Strain s2753

Bacterial strain s2753 was isolated from a mussel surface collected in the tropical Pacific (9.1° S 156.8° E) during the Danish Galathea 3 expedition [3]. S2753 was assigned to the *Vibrionaceae* and sequence similarity of household genes identified it as a *Photobacterium halotolerans* as previously described [3,4,5].

### 3.2. Isolation and Structure Elucidation of Four New Ngercheumicins

S2753 was cultured in 10 L glass fermentors in 5 × 4 L SSS containing 0.4% glucose and 0.3% casamino acids in (25 °C, 72 h, 100 rpm). The liquid culture was centrifuged (15 min, 3500 × *g*) to isolate the pellet from the broth and Diaion HP20SS (12 g L^−1^) which was used to extract the bioactive compounds holomycin and solonamides A–B as previously described [4,5]. The pellet was extracted with 1 L 1:9 (v/v) MeOH/EtOAc (25 °C, 24 h, 100 rpm) and filtered off through a Watman 1 filter. The pellet extract was concentrated on a rotary evaporator and absorbed onto 5 g Isolute diol (Biotage, Uppsala, Sweden) for dry loading onto a 50 g SNAP column packed with Isolute diol and eluted on an Isolera automated flash system (Biotage, Uppsala, Sweden) using solvents ranging from heptane, dichloromethane, EtOAc to pure MeOH (30 mL min^−1^, 72 min). A total of 33 fractions were collected and subjected to LC-UV/MS. Fractions 7 to 10 (dichloromethane/EtOAc) were pooled and absorbed onto 1.5 g Isolute diol and further fractionated on a 10 g diol column run by gravity with heptanes, dichloromethane, EtOAc and MeOH as above. This yielded 10 fractions (again subjected to LC-UV/MS) of which fractions 6 and 7 (10%–30% MeOH in EtOAc) were purified on a Luna II column (5 μm C_18_, 250 × 10 mm ID, Phenomenex) in a Gilson 322 liquid chromatograph with a 215 liquid handler/injector (BioLab, Risskov, Denmark) going from 70% to 100% aqueous MeCN, 20 mM formic acid, over 10 min followed by 6 min isocratic elution. This yielded 12 fractions of which pure compounds were obtained directly: Ngercheumicin F (0.5 mg), Ngercheumicin G (1.0 mg), Ngercheumicin H (0.5 mg), and Ngercheumicin I (1.1 mg). Selected chromatograms are available in the Supplementary Information.

LC-UV/MS analyses were performed on an Agilent 1100 HPLC system with a diode array detector coupled to an LCT TOF mass spectrometer (Micromass, Manchester, UK) using a Z-spray ESI source. Separation was performed at 40 °C with a Luna II C_18_ column (50 × 2 mm ID, 3 μm, Phenomenex, Torrance, CA, USA), applying a linear gradient of 15%–100% aqueous MeCN, 20 mM formic acid (LC-MS-grade), over 20 minutes at a flow rate of 0.3 mL/min. MS experiments were performed in ESI^+^ with a data acquisition range of *m/z* 100–2000. Accurate masses of ammonium adducts were measured for Ngercheumicin F (*m/z*: [M + NH_4_]^+^ 870.5979, calculated for C_43_H_80_N_7_O_11_ as 870.5916), ngercheumicin G (*m/z*: [M + NH_4_]^+^ 872.6206, calculated for C_43_H_82_N_7_O_11_ as 872.6072), ngercheumicin H (*m/z*: [M + NH_4_]^+^ 898.6359, calculated for C_45_H_84_N_7_O_11_ as 898.6229), and ngercheumicin I (*m/z*: [M + NH_4_]^+^ 900.6450, calculated for C_45_H_86_N_7_O_11_ as 900.6385). The [M + H]^+^ adducts were reported in Section 2.1.

To solve the absolute configuration of the amino acids Marfey’s method was applied: 100 μg of each cyclodepsipeptide was subjected to acid hydrolysis (200 μL 6 M HCl, 110 °C, 20 h), redissolved in 50 μL water and added 20 μL 1 M aqueous NaHCO_3_, then derivatised with 100 μL 1% w/v Marfey’s reagent in acetone (1-fluoro-2,4-dinitrophenyl-5-l-alanine amide, FDAA, Sigma-Aldrich, St. Louis, MO, USA) at 40 °C for 1 h as described by Bonnard *et al.* [15]. The reaction mixtures were neutralised with 10 μL 2 M aqueous HCl and diluted with 820 μL MeOH. Pure amino acid standards were derivatised by the same procedure using 50 μL 50 mM aqueous amino acid solution. Ultra-high performance liquid chromatography-diode array (UHPLC-DAD) separation and detection of the amino acid derivatives was done on a Dionex RSLC Ultimate 3000 (Dionex, Sunnyvale, CA, USA) equipped with a diode array detector. The separation was done in a Kinetex C_18_ column (150 × 2.10 mm, 2.6 μm, Phenomenex) at 60 °C with a flow rate of 0.8 mL min^−1^ using two different linear gradient methods. Method A included all l- and d-amino acids in the structure, whereas method B was run with shallow gradient and included l- and d-*allo*-Thr but not l- and d-Leu. This was an attempt to distinguish derivatives of d-Ser, l-Thr and l-*allo*-Thr which had very similar chromatographic properties on the column.

Method A: From 8% to 15% aqueous MeCN, 0.65 mM TFA, over 22 min followed by an increase from 15% to 100% for 8.5 min. Retention times for the FDAA-amino acid derivatives were: l-Ser (4.21 min), l-Thr (5.46 min), d-Ser (5.56 min), d-Thr (12.38 min), l-Leu (25.23 min), d-Leu (26.00 min). Unreacted FDAA eluted at 11.0 min.

Method B: From 8% to 10% aqueous MeCN, 0.65 mM TFA, over 25 min followed by an increase from 10% to 100% over 5.5 min. Retention times for the FDAA-amino acid derivatives were: l-Ser (4.46 min), l-Thr (5.90 min), d-Ser (5.97 min), l-*allo*-Thr (6.15 min), d-*allo*-Thr (10.28 min), d-Thr (15.82 min). Unreacted FDAA eluted at 10.4 min.

NMR spectra were recorded on a Bruker Avance 800 MHz spectrometer equipped with a 5 mm TCI Cryoprobe using standard pulse sequences. The NMR data used for the structural assignments were acquired in DMSO-*d*_6_ (δ_H_ 2.49 ppm and δ_C_ 39.5 ppm). ^1^H and ^13^C NMR spectra are available in the Supplementary Information.

### 3.3. Antivirulence Activity Testing and Northern Blotting

The *S. aureus lacZ* reporter assays were performed as described by Nielsen *et al.* [19]. Ngercheumicin F, G, H, and I were dissolved in DMSO, and DMSO and H_2_O was included as negative controls in the assay. Pictures were taken after 11, 13 and 34 h of incubation for the *rnaIII*-, *hla*- and *spa*-reporter strains respectively (Supplementary Information).

RNA for Northern blotting was purified from USA300 (FPR3757) samples from cultures grown in 100 mL Erlenmeyer flasks containing 10 mL Tryptone Soya Broth (TSB, Oxoid, Greve, Denmark) shaking vigorously (200 rpm) in a water bath at 37 °C. Ngercheumicins (5 µg mL^−1^ and 20 µg mL^−1^) and DMSO (control) were added at OD_600_ = 0.4, and samples were taken at OD_600_ = 0.8 and 3.0. Northern blotting was performed as previously described [5] using an RNAIII-probe constructed using primer *rnaIII* forward (5′-GGG GAT CAC AGA GAT GTG ATG-3′), and *rnaIII* reverse (5′-GGG CAT AGC ACT GAG TCC AAG G-3′)(TAG Copenhagen A/S, Frederiksberg, Denmark).

## 4. Conclusions

Four new ngercheumicins were isolated on the mg-scale and their structure elucidated; however complete stereochemical assignments were not obtained. Although 20 L of bacterial culture was extracted, low isolated yields restricted the possibilities in both the structure elucidation and in the assessment of biological properties. Ngercheumicins were found to inhibit transcription of the regulatory *rnaIII* in *S. aureus*, the effector molecule of the *agr* QS system. These findings will aid in the future work to understand quorum sensing in bacteria, as more structural knowledge about QS inhibitors is valuable in the design of novel inhibitors. The cyclodepsipeptides isolated from this marine *Photobacterium* have some resemblance to the AIPs of *S. aureus*, and it can be speculated as to whether these molecules are a new class of peptide signaling molecules in the marine environment.

## Supplementary Files

Supplementary File 1 Supplementary Information (PDF, 1327 KB)
