# Supplementary material for: Identification of Four New agr Quorum Sensing-Interfering Cyclodepsipeptides from a Marine Photobacterium
Source: Mar Drugs. 2013 Dec 12;11(12):5051–62. doi: 10.3390/md11125051 (PMC3877902; doi:10.3390/md11125051)
Supplement: Supplementary File 1 — Supplementary Information (PDF, 1327 KB) [file marinedrugs-11-05051-s001.pdf]

# Supplementary Information


**Figure S1.** HPLC-UV chromatogram from final RP purification step (Gilson).
**Figure S2.** LC-UV-MS diode array chromatograms for ngercheumicin F–I.
**Figure S3.** Selected regions of $ESI^+$ mass spectra for purified ngercheumicin F–I.
**Figure S4.** $^1$H NMR spectrum for ngercheumicin F.
**Figure S5.** DQF-COSY for ngercheumicin F.
**Figure S6.** Multiplicity edited gHSQC for ngercheumicin F.
**Figure S7.** gHMBC for ngercheumicin F.
**Figure S8.** $^1$H NMR spectrum for ngercheumicin G.
**Figure S9.** $^{13}$C NMR spectrum for ngercheumicin G.
**Figure S10.** DQF-COSY for ngercheumicin G.
**Figure S11.** Multiplicity edited gHSQC for ngercheumicin G.
**Figure S12.** gHMBC for ngercheumicin G.
**Figure S13.** $^1$H NMR spectrum for ngercheumicin H.
**Figure S14.** $^{13}$C NMR spectrum for ngercheumicin H.
**Figure S15.** DQF-COSY for ngercheumicin H.
**Figure S16.** Multiplicity edited gHSQC for ngercheumicin H.
**Figure S17.** gHMBC for ngercheumicin H.
**Figure S18.** $^1$H NMR spectrum for ngercheumicin I.
**Figure S19.** $^{13}$C NMR spectrum for ngercheumicin I.
**Figure S20.** DQF-COSY for ngercheumicin I.
**Figure S21.** Multiplicity edited gHSQC for ngercheumicin I.
**Figure S22.** gHMBC for ngercheumicin I.
**Figure S23.** Key HMBC and H2BC correlations in the fatty acid chain of Ngercheumicin F.
**Figure S24.** Northern blot results for ngercheumicin F–I.
**Figure S25.** Plate assay results in the colorimetric *S. aureus lacZ* reporter assay against *S. aureus* 8325-4.
**Table S1.** NMR table for ngercheumicin F.
**Table S2.** NMR table for Ngercheumicin G.
**Table S3.** NMR table for Ngercheumicin H.
**Table S4.** NMR table for Ngercheumicin I.

**Figure S1.** Detector response from HPLC purification of ngercheumicins, where fractions 6, 8, 9 and 11 gave ngercheumicin F, G, H and I, respectively. Fraction size is not shown, as these are pooled fractions from the automatic fractionation. Fraction 7 and 10 contains isomers of ngercheumicin F and H.

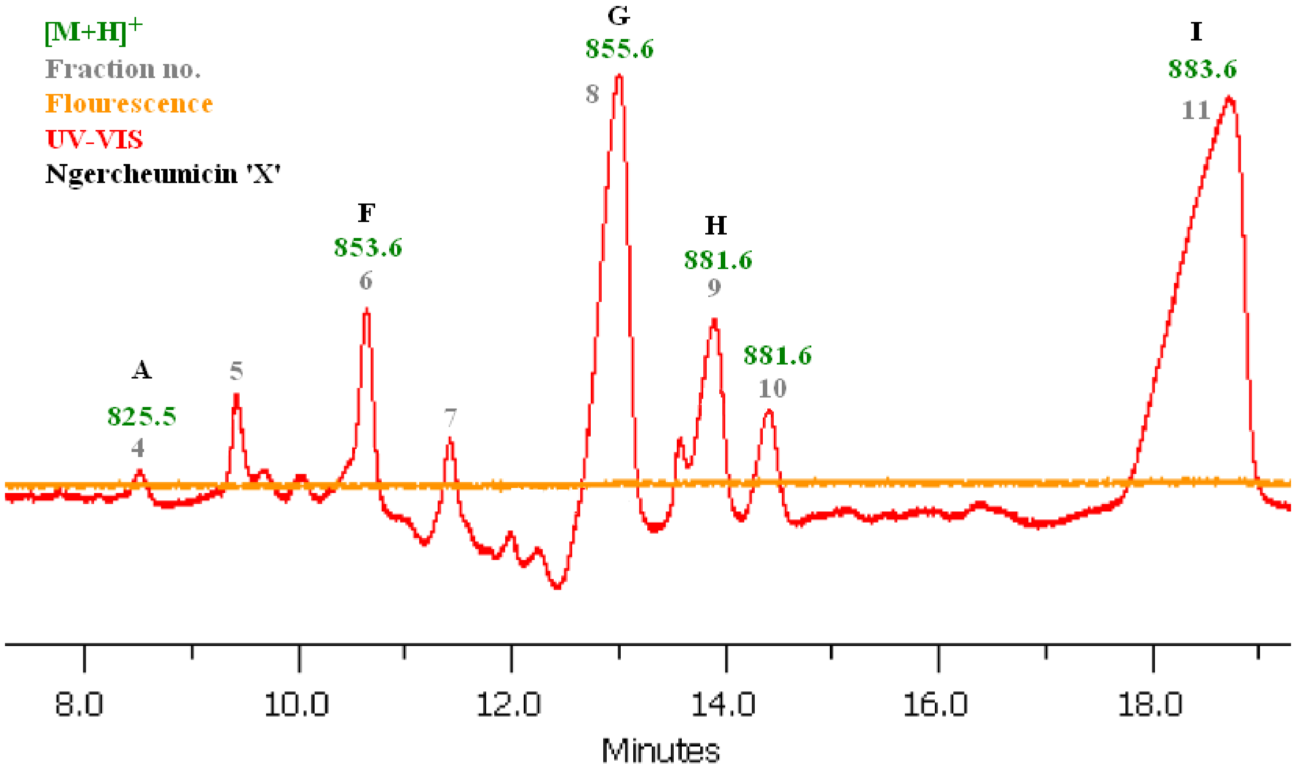

**Figure S2.** Diode array detected (200–700 nm) chromatograms for purified samples of ngercheumicin F, G, H and I (from the top).

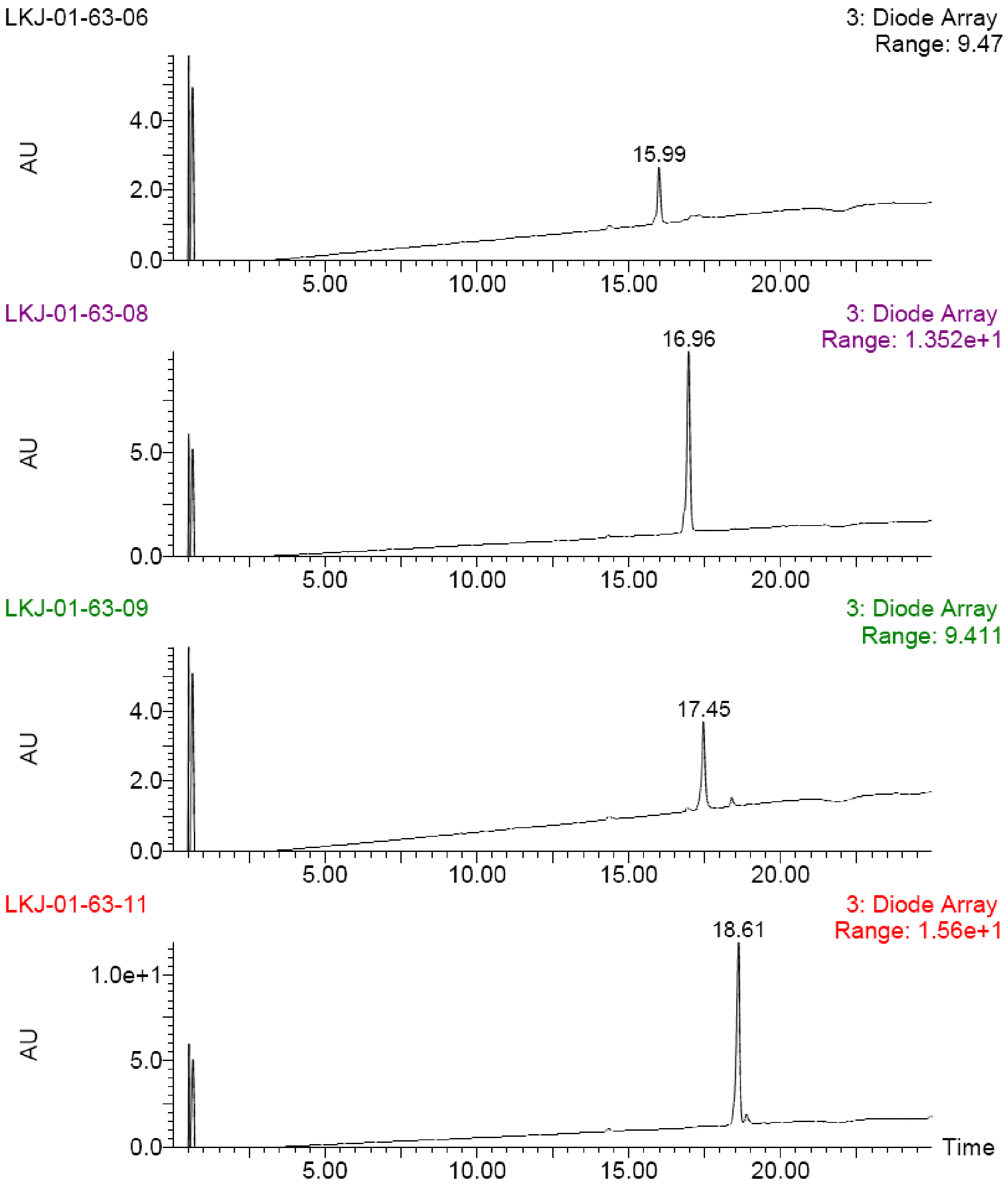

**Figure S3.** Selected region of the total ion chromatograms (TIC) of ngercheumicin F, G, H and I (from the top), showing $[M + H]^+$, $[M + NH_4]^+$ and weak $[M + Na]^+$ adducts. Outside the range are also $[2M + NH_4]^+$ at *m/z* 1723–1784.

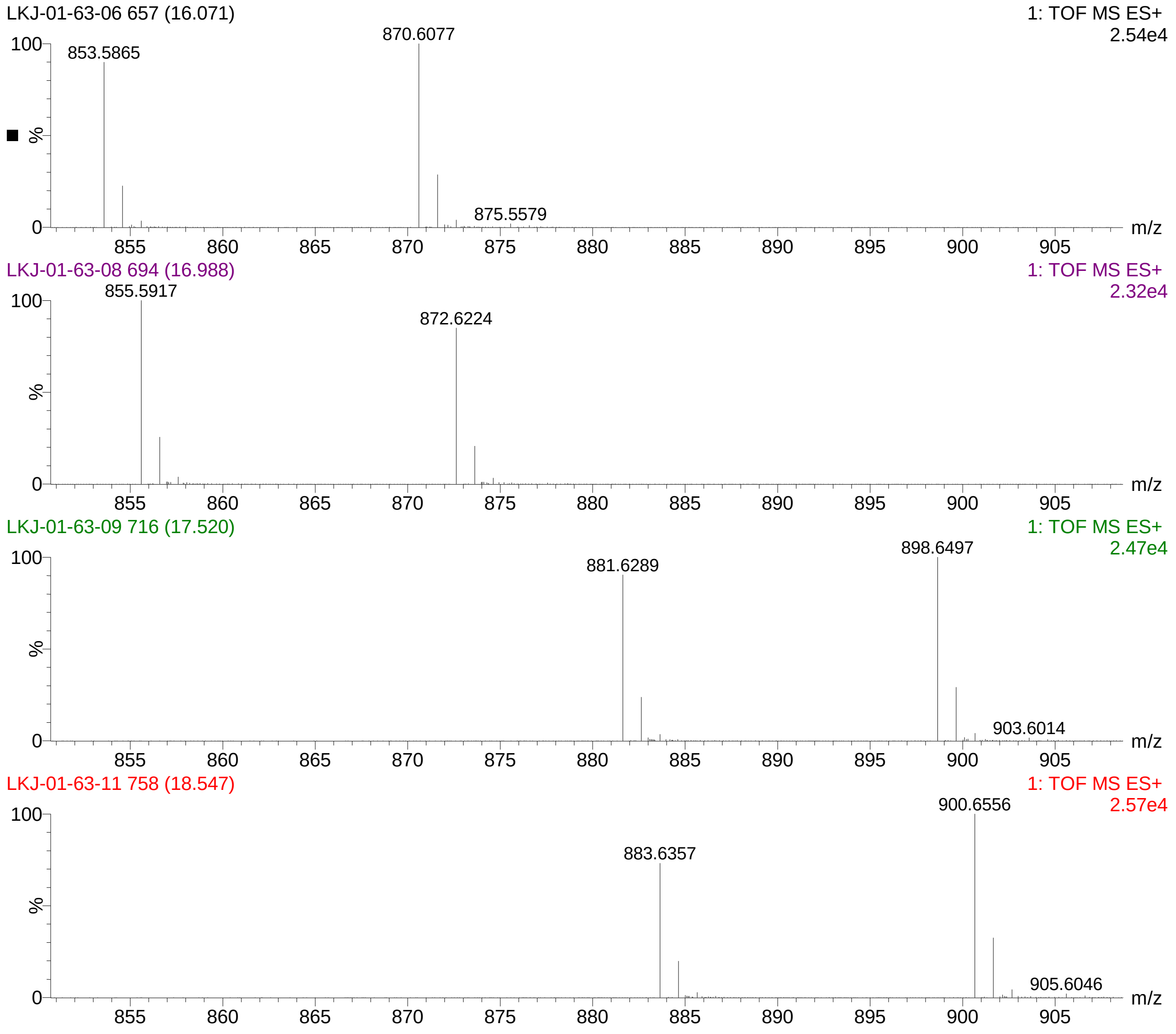

**Figure S4.** $^{1}$H spectrum for ngercheumicin F.

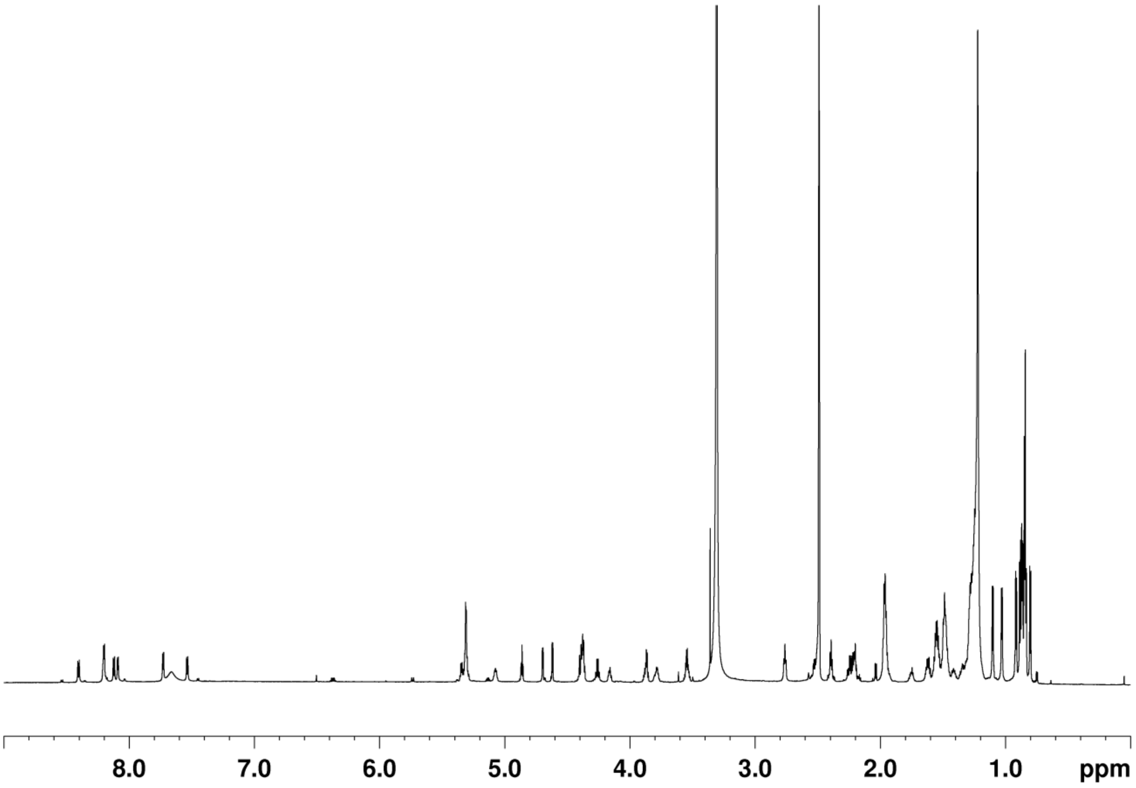


**Figure S5.** DQF-COSY for ngercheumicin F.

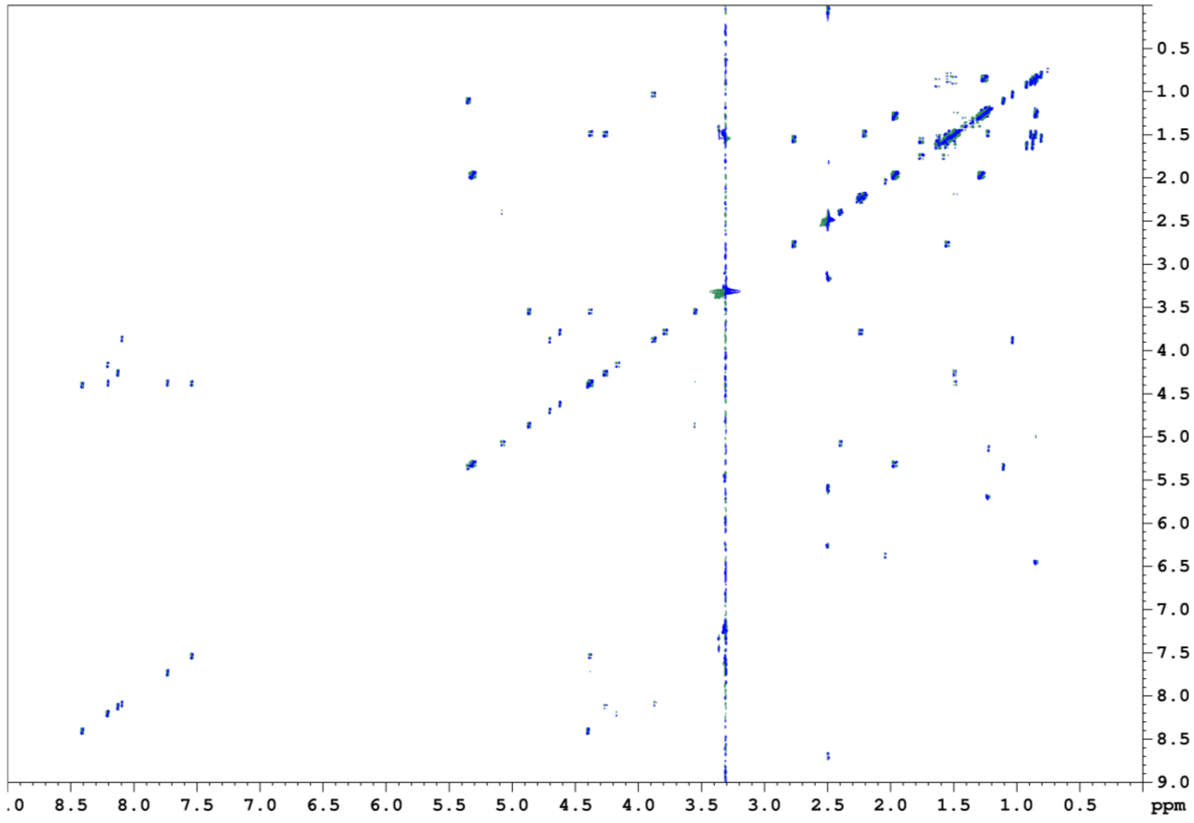

**Figure S6.** Multiplicity edited gHSQC for ngercheumicin F.

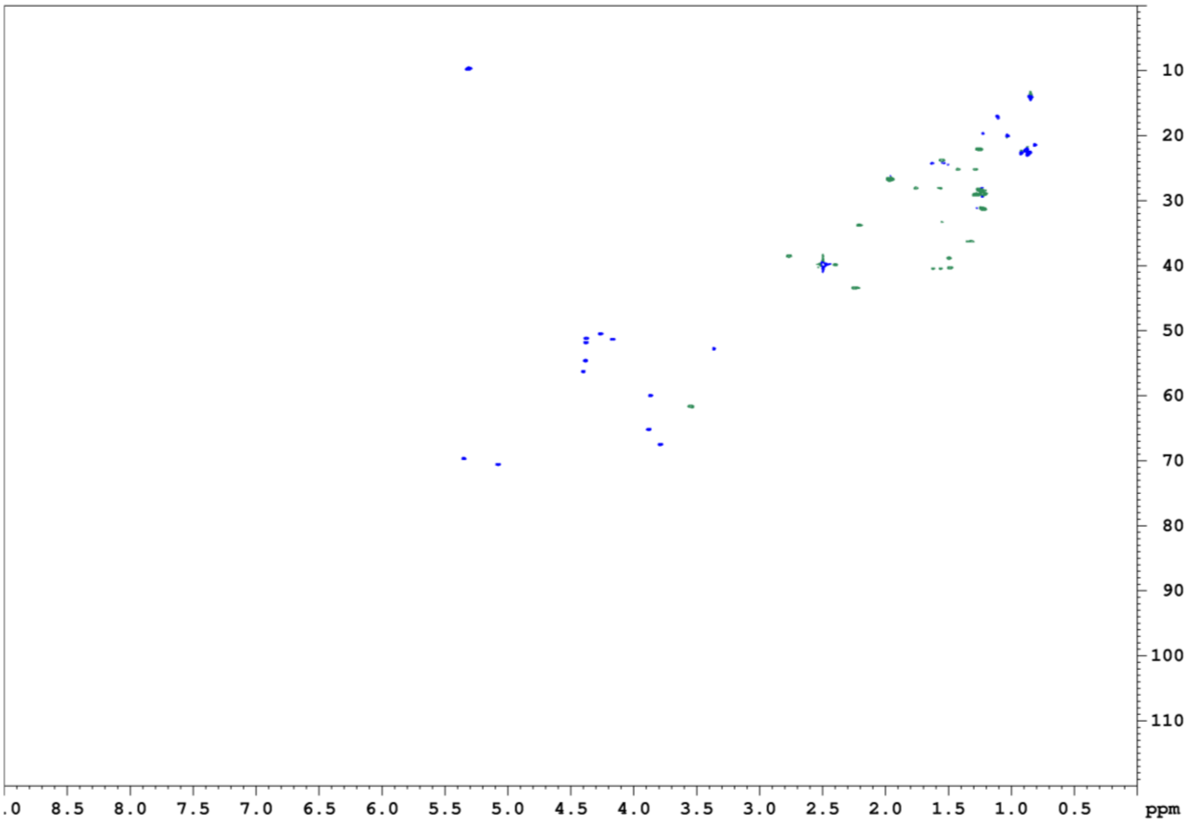


**Figure S7.** gHMBC for ngercheumicin F.

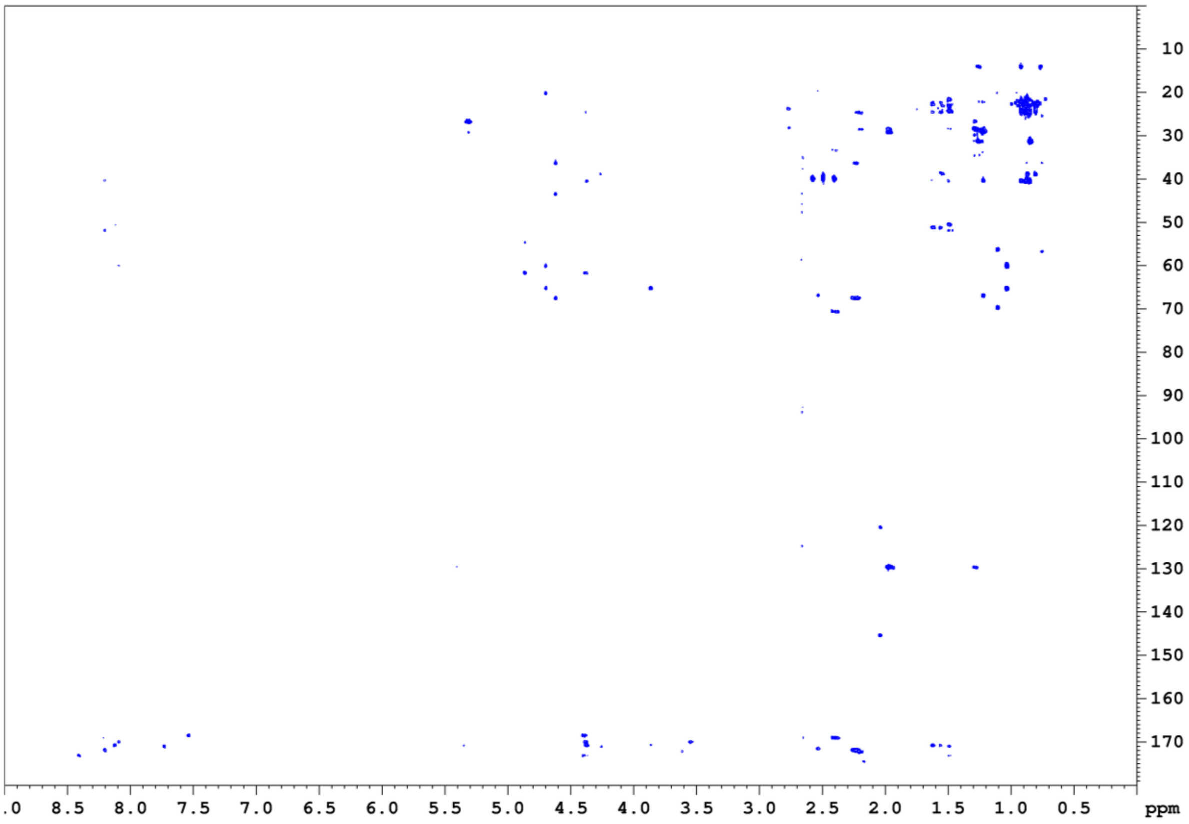

**Figure S8.** $^{1}$H spectrum for ngercheumicin G.

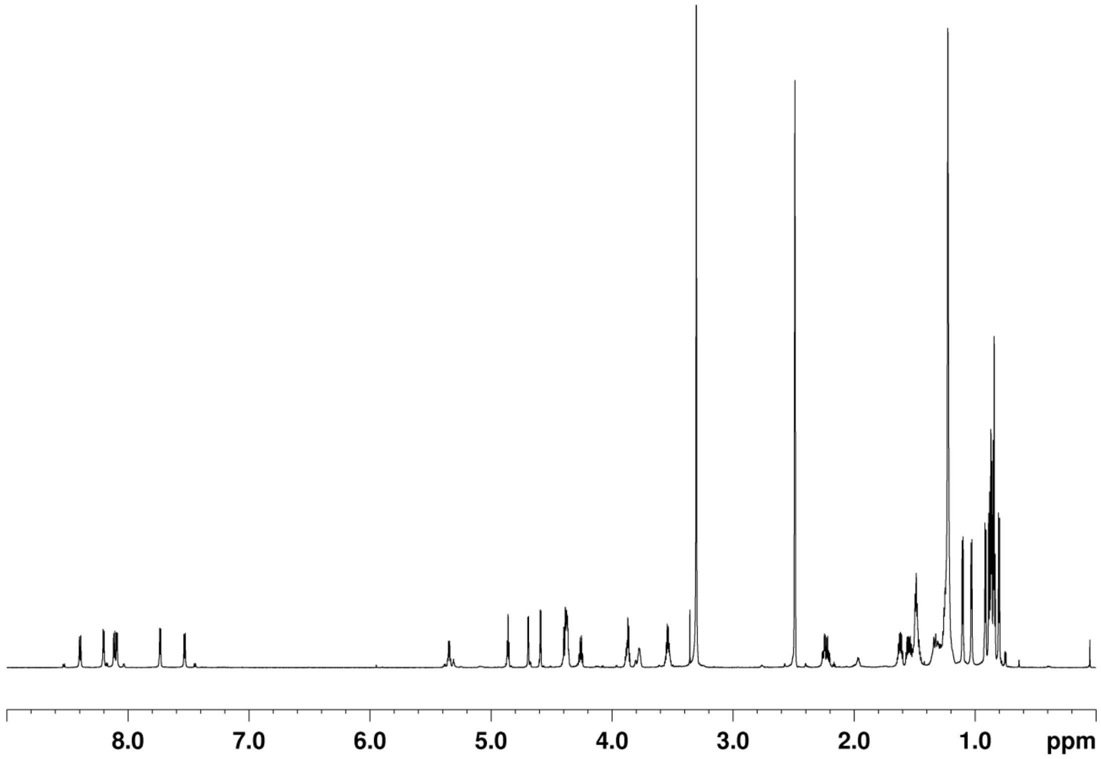


**Figure S9.** $^{13}$C spectrum for ngercheumicin G.

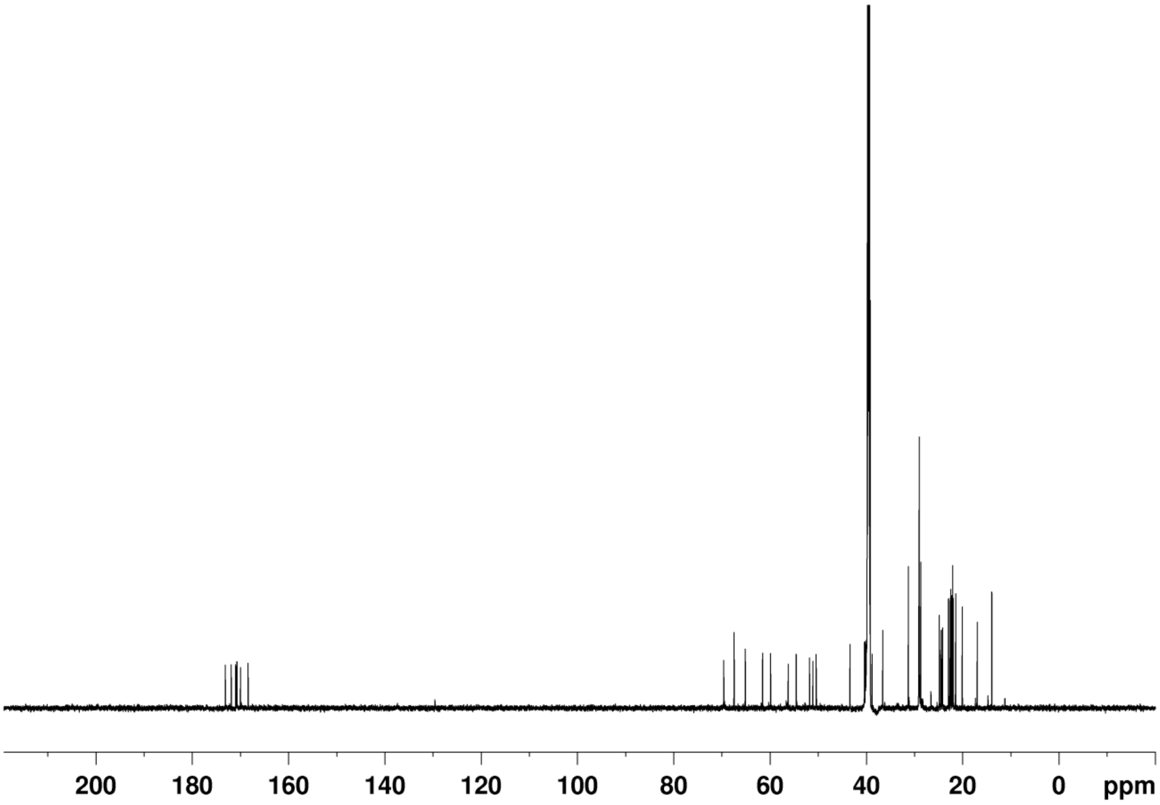

**Figure S10.** DQF-COSY for ngercheumicin G.

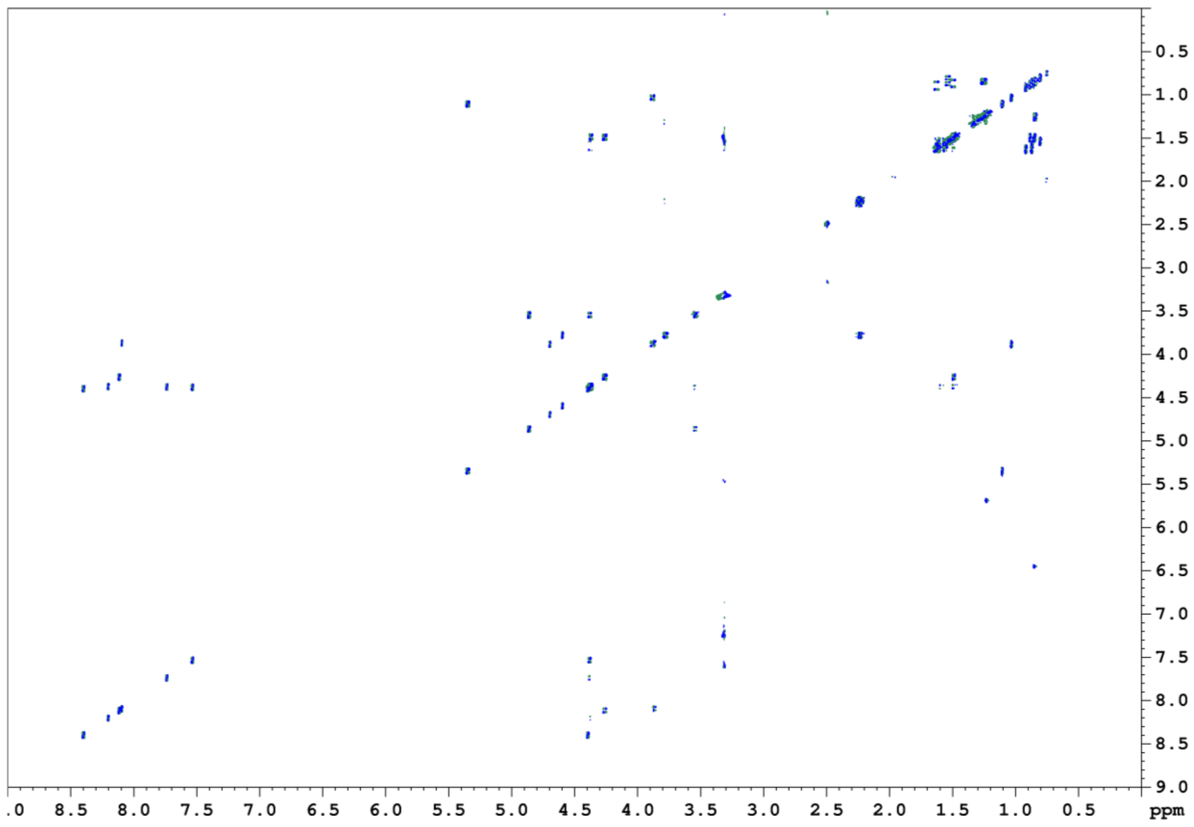


**Figure S11.** Multiplicity edited gHSQC for ngercheumicin G.

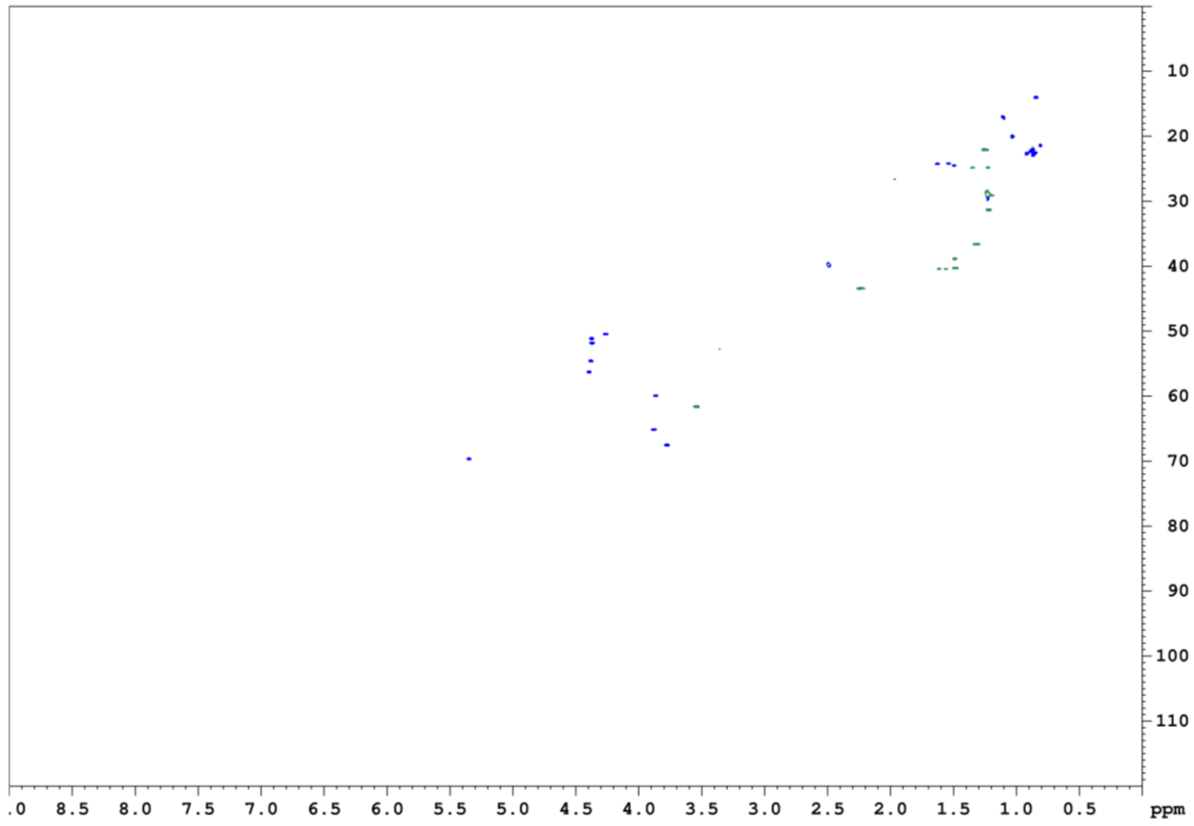

**Figure S12.** gHMBC for ngercheumicin G.

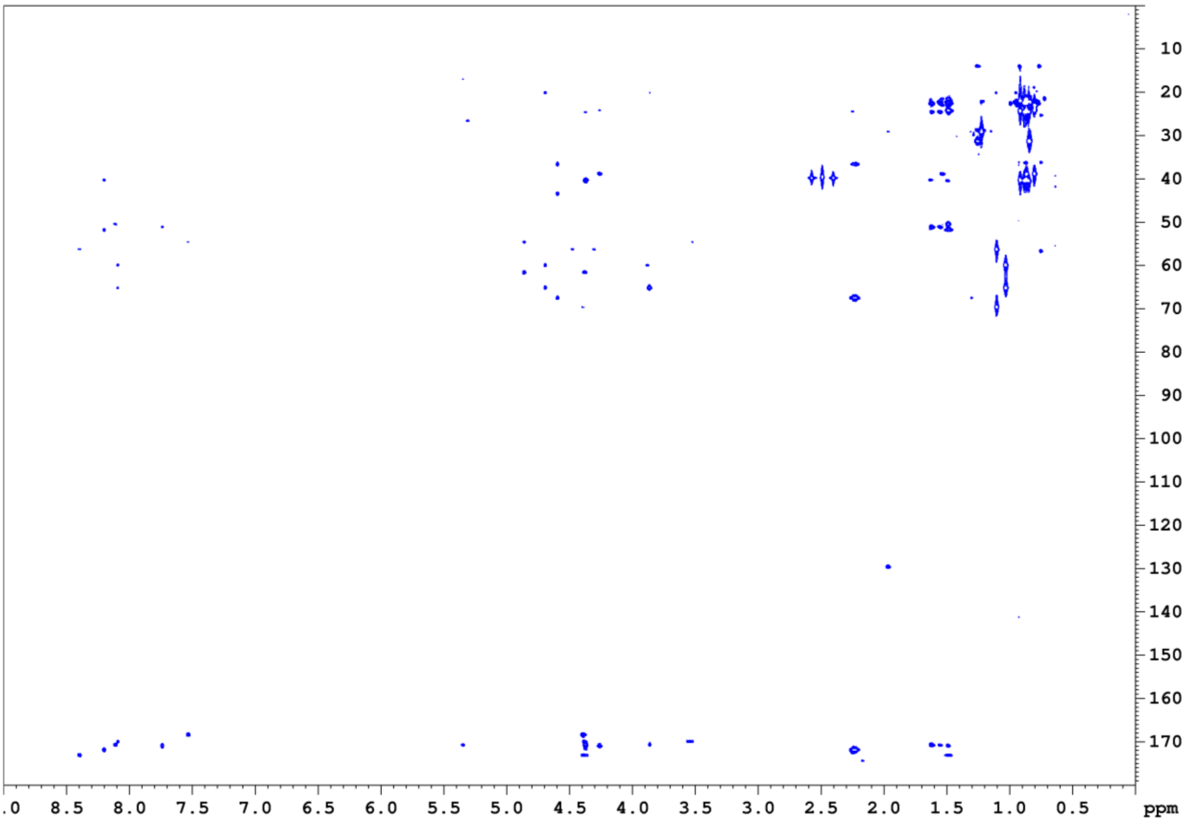


**Figure S13.** $^{1}$H spectrum for ngercheumicin H.

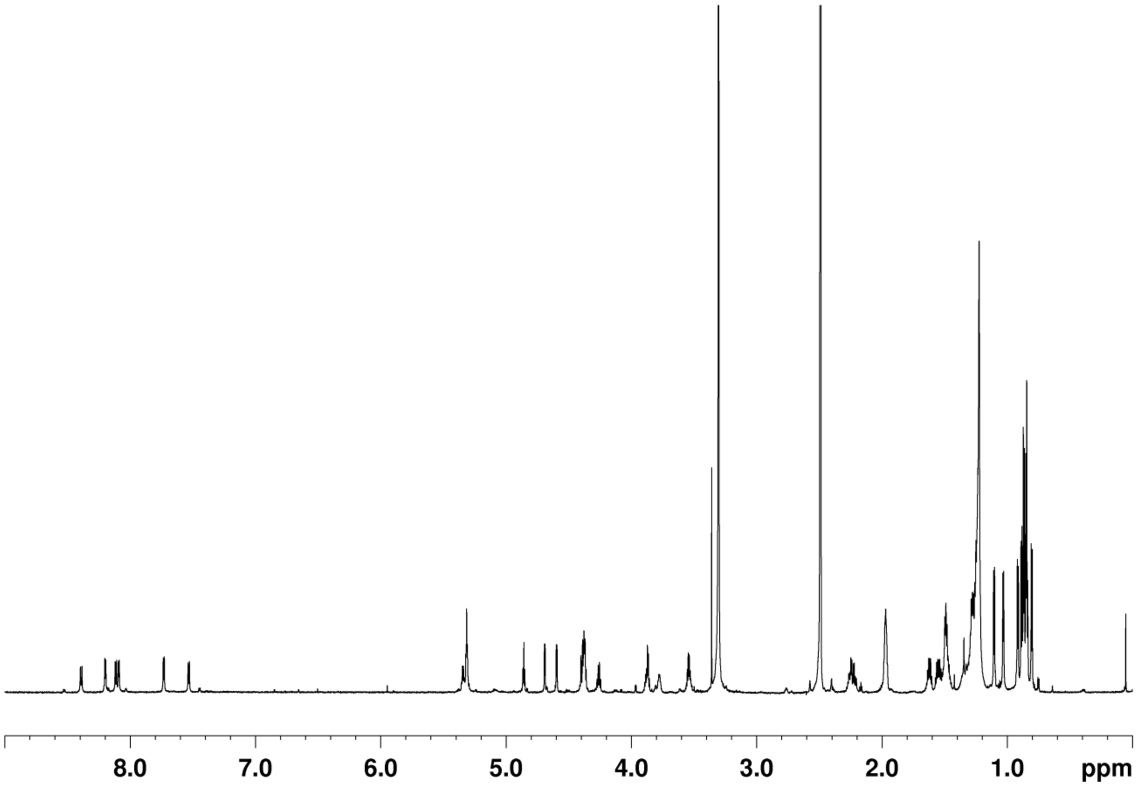

**Figure S14.** $^{13}C$ spectrum for ngercheumicin H.

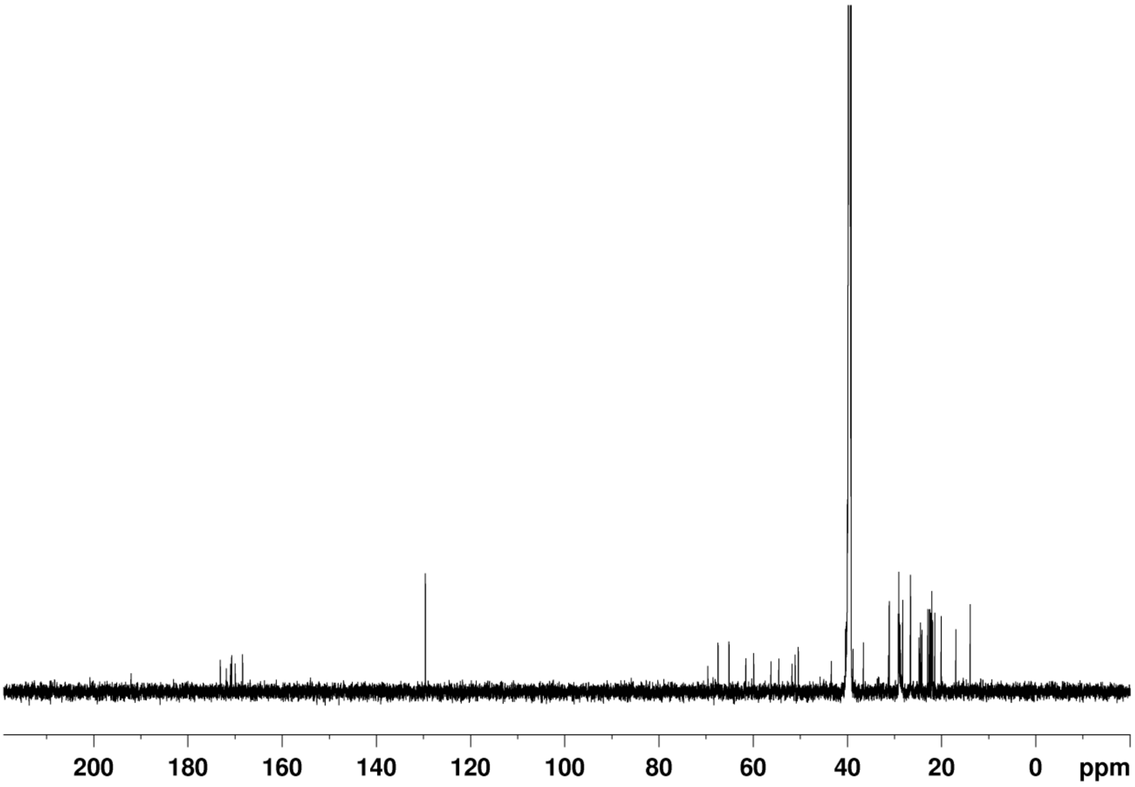


**Figure S15.** DQF-COSY for ngercheumicin H.

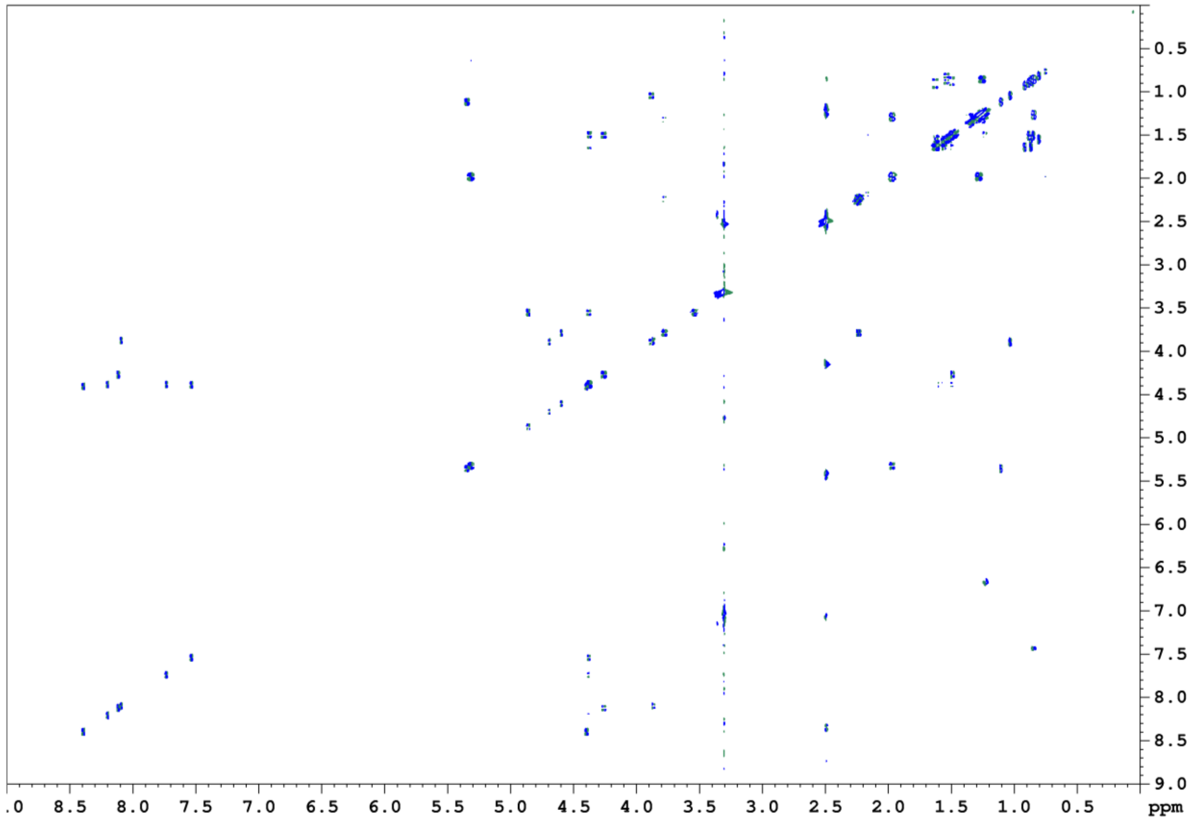

**Figure S16.** Multiplicity edited gHSQC for ngercheumicin H.

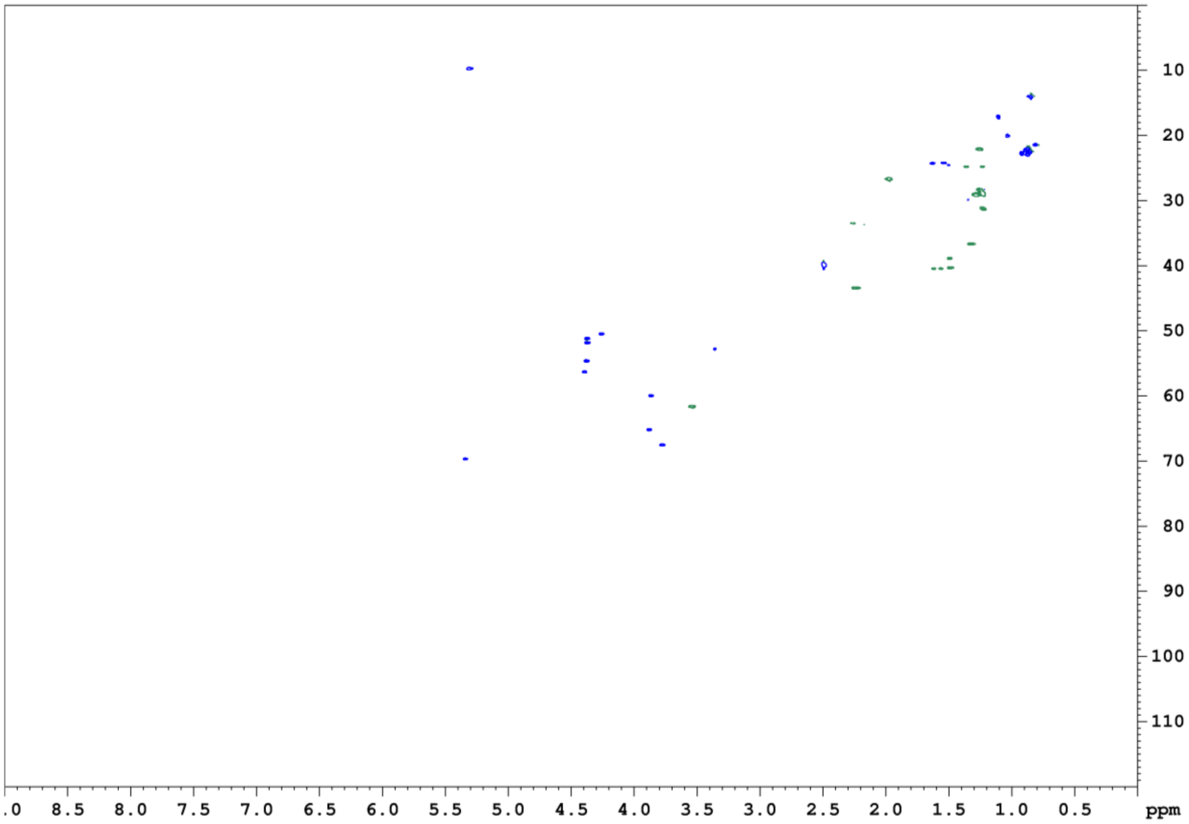


**Figure S17.** gHMBC for ngercheumicin H.

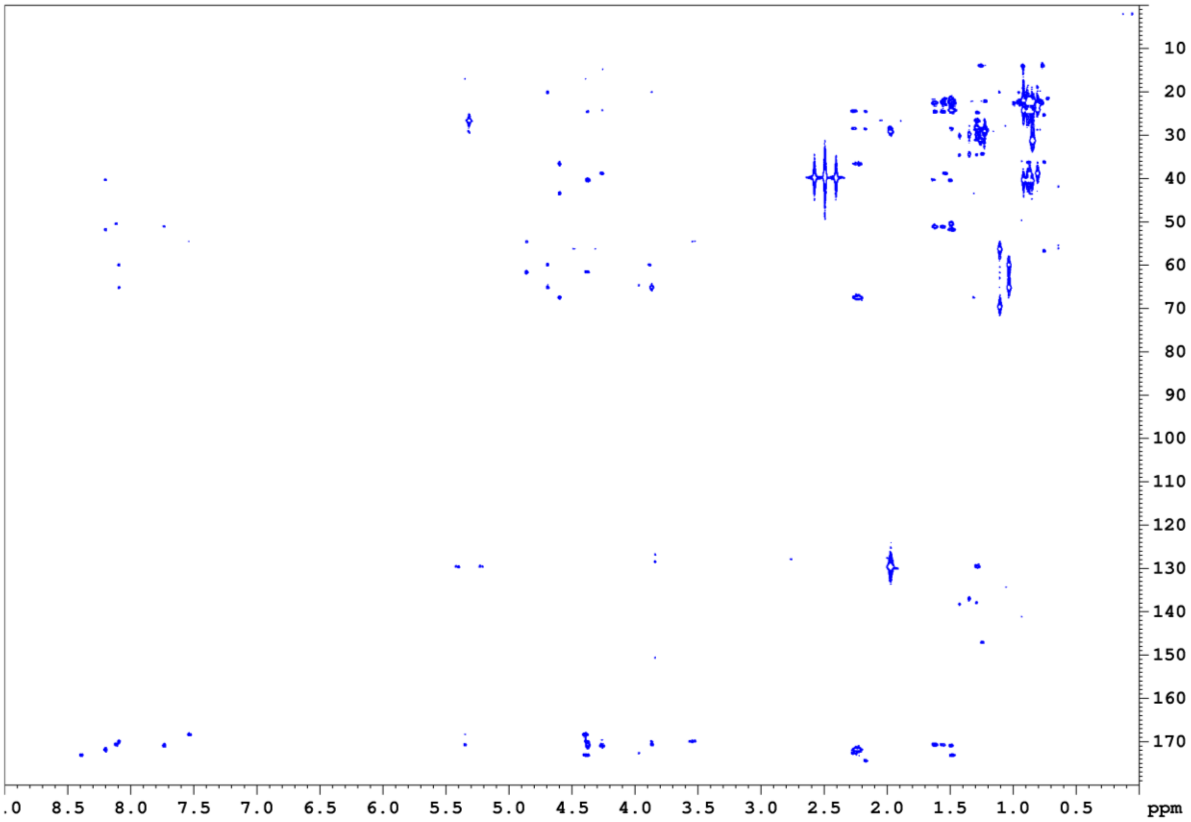

**Figure S18.** $^{1}$H spectrum for ngercheumicin I.

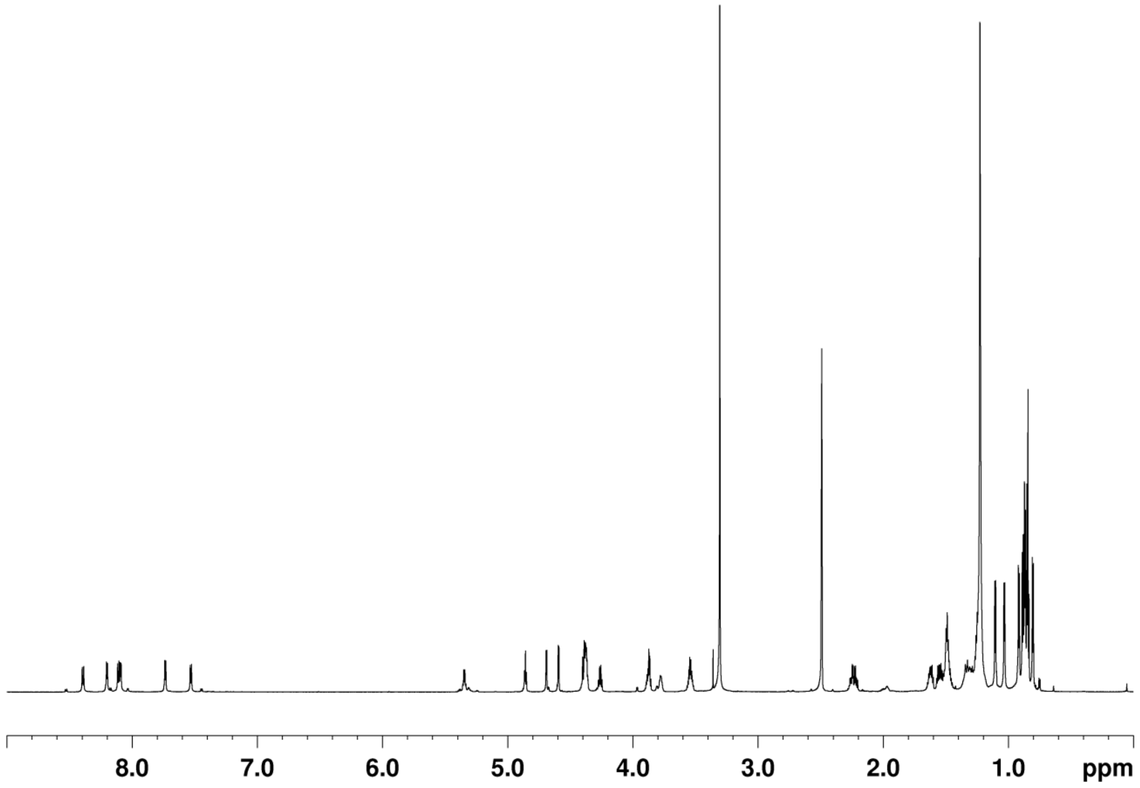


**Figure S19.** $^{13}$C spectrum for ngercheumicin I.

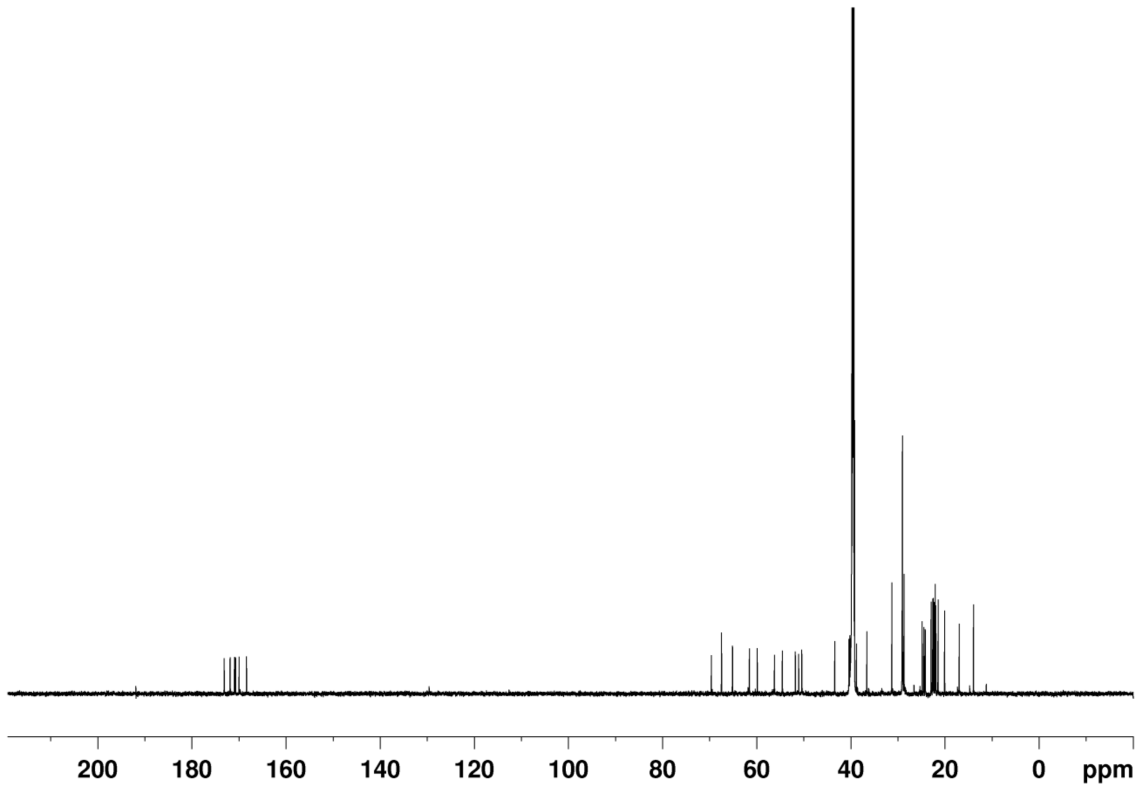

**Figure S20.** DQF-COSY for ngercheumicin I.

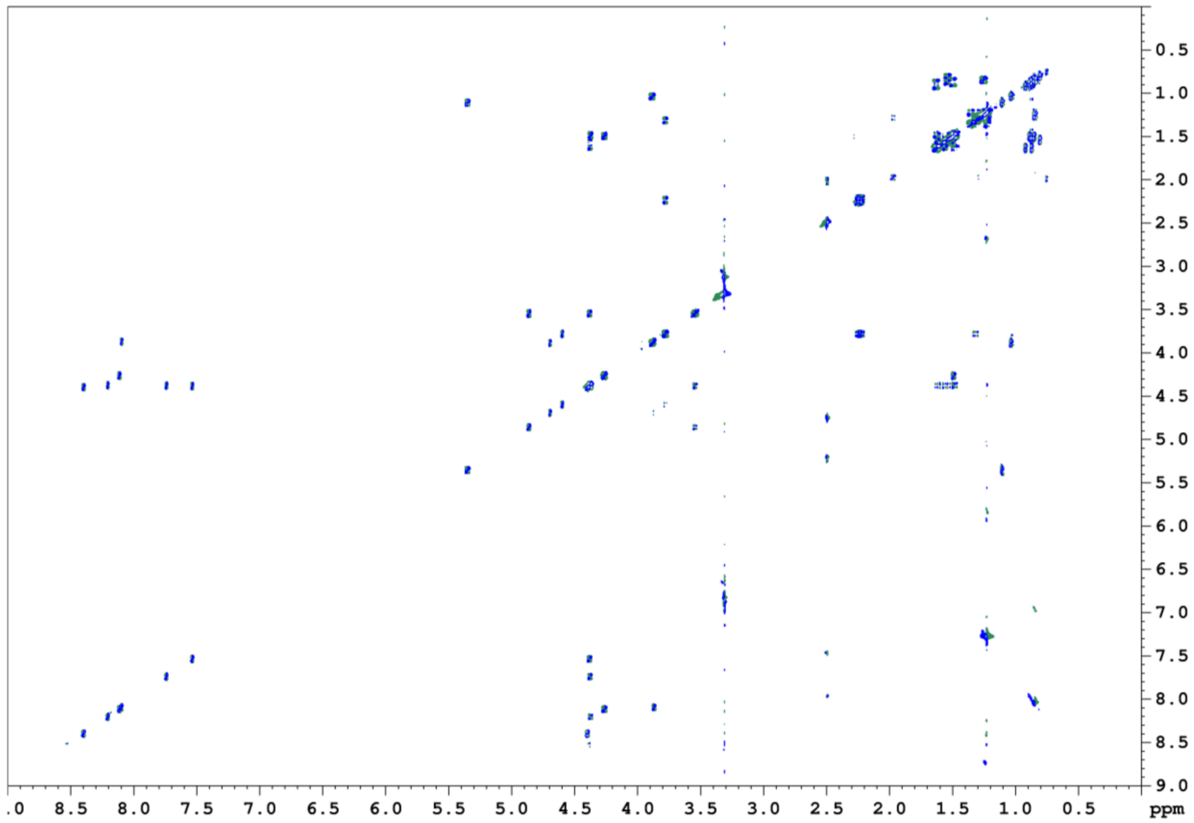


**Figure S21.** Multiplicity edited gHSQC for ngercheumicin I.

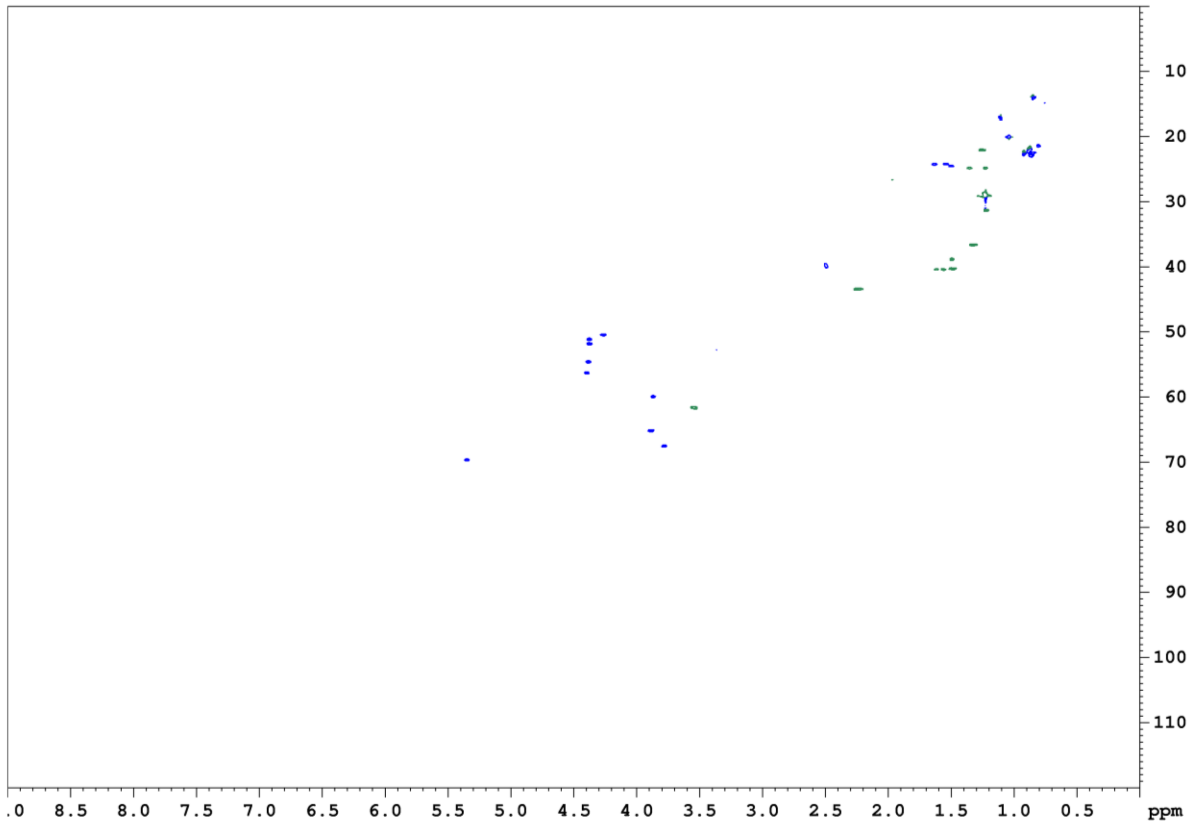

**Figure S22.** gHMBC for ngercheumicin I.

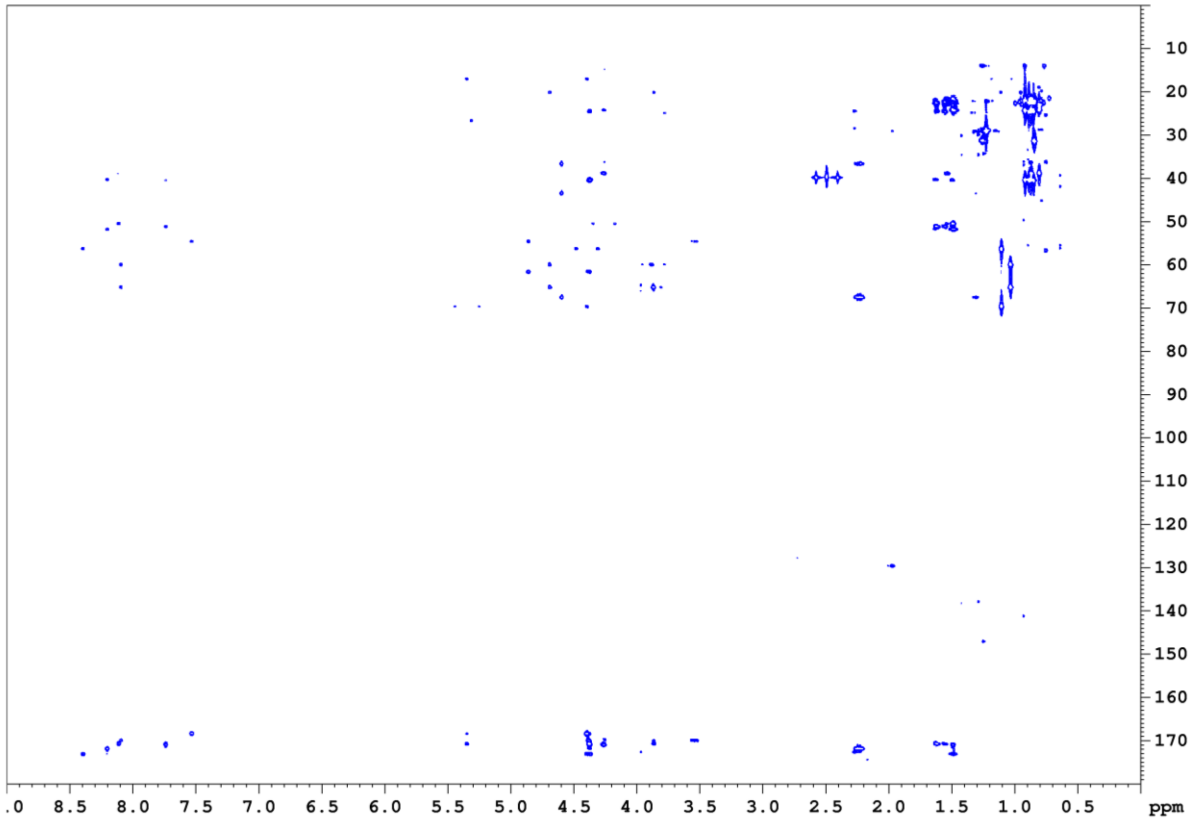

**Figure S23.** Key HMBC and H2BC correlations in the fatty acid chain of Ngercheumicin F.

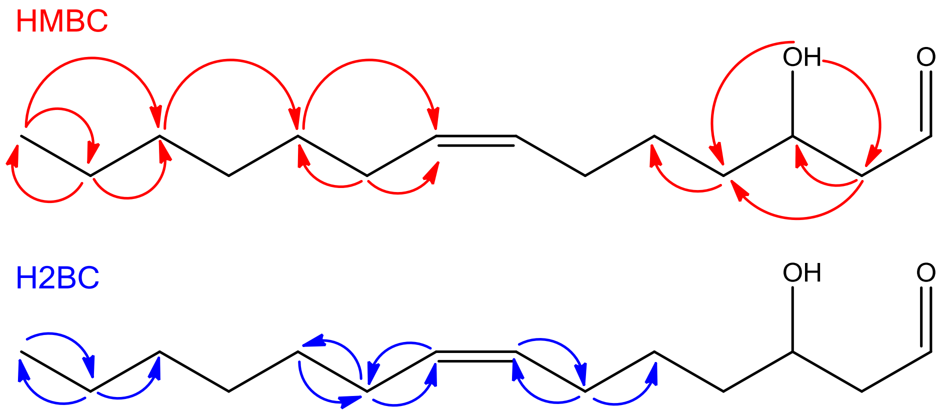

**Figure S24.** Northern blot results for ngercheumicin F, G, H and I in DMSO at 5 µg/mL (≈6 µM) and 20 µg/mL (≈23 µM) against *S. aureus* USA300/FPR3757 at $OD_{600}$ = 0.8 and $OD_{600}$ = 3.0. DMSO is used as negative control.

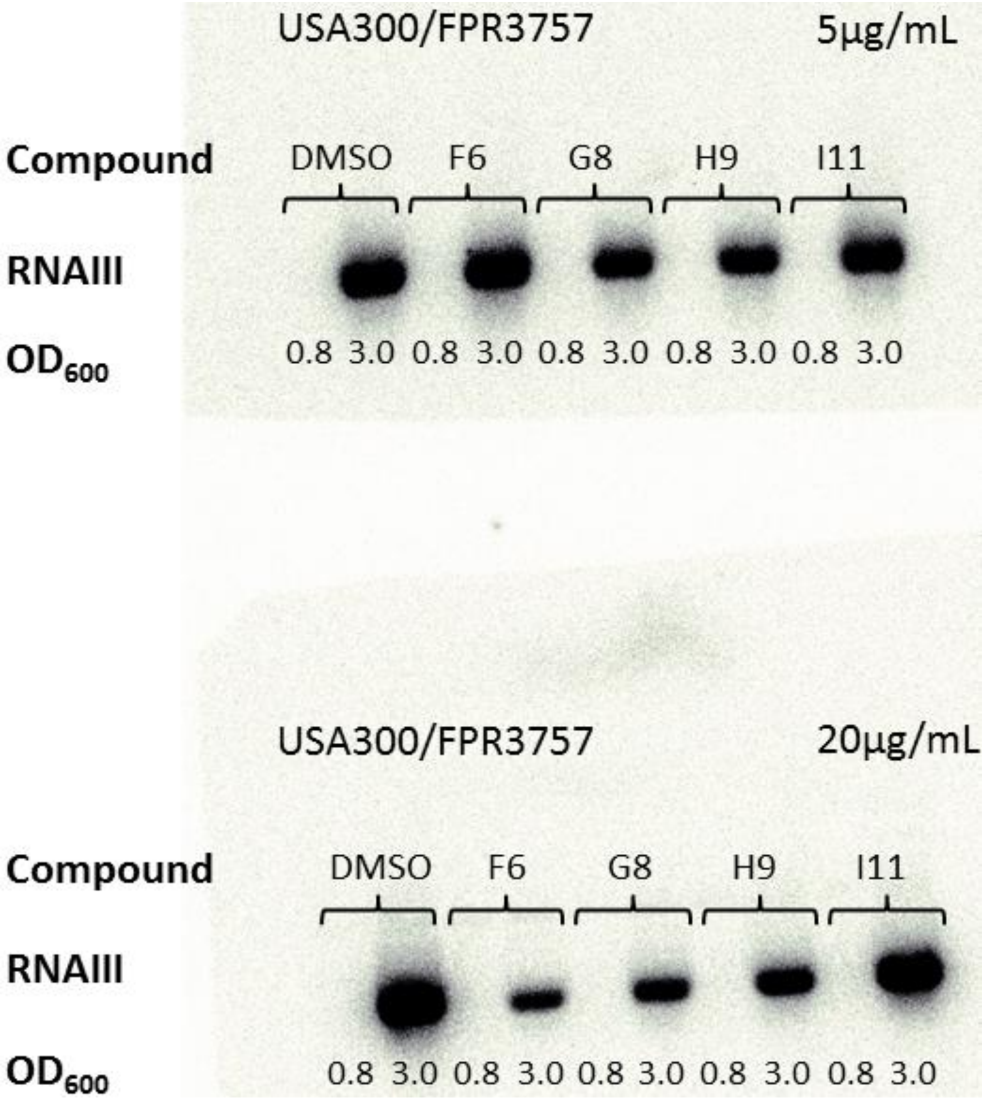

**Figure S25.** Plate assay results in the colorimetric *S. aureus lacZ* reporter assay with ngercheumicins F, G, H and I in DMSO at 1 mg/mL against *S. aureus* 8325-4. DMSO and water is used as negative controls. Inhibitors of the *agr* locus enhance transcription of *spa* and reduce expression of *hla* and *rnaIII*.

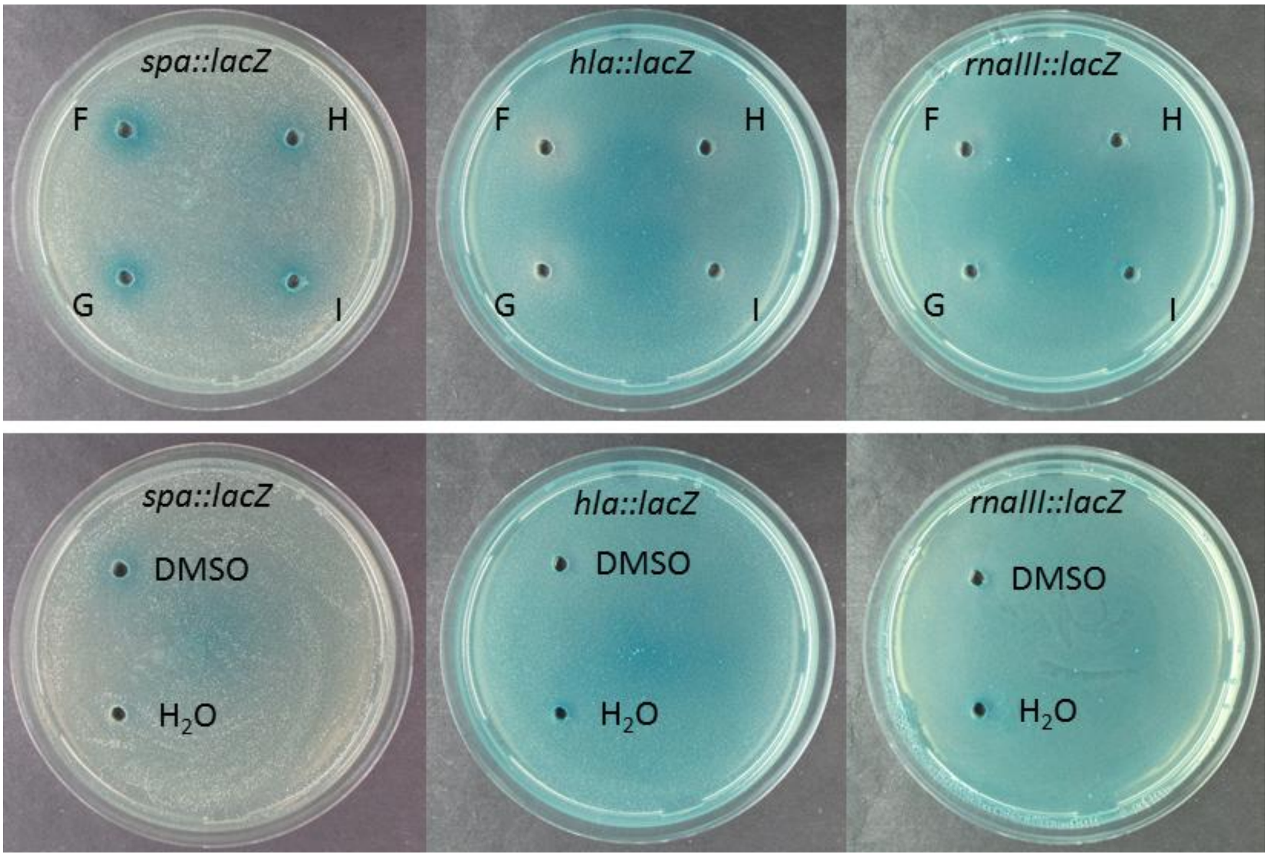


**Table S1.** NMR table for Ngercheumicin F.

| Position, Type | $\delta_H$ (ppm), #H, Multiplicity, *J* (Hz) | $\delta_C$ (ppm) | HMBC Connectivity | H2BC Connectivity |
|---|---|---|---|---|
| *Leu*[1] | | | | |
| 1—CO | – | 170.8 | – | |
| 2—$CH_\alpha$ | 4.37, 1H, m | 51.1 | 1, 3 | |
| 3—$CH_\beta$ | 1.62, 1H, m | 40.4 | 1, 2, 4, 5, 6 | |
| | 1.55, 1H, m | | | |
| 4—$CH_\gamma$ | 1.49, 1H, m | 24.5 | 3 | |
| 5—$CH_{\delta,1}$ | 0.88, 3H, d, 6.5 | 22.3 | 3, 4, 6 | 4 |
| 6—$CH_{\delta,2}$ | 0.85, 3H, d, 6.5 | 22.5 | 3, 4, 5 | 4 |
| 7—NH | 7.73, 1H, d, 6.5 | – | (2), 8 | 2 |
| *Leu*[2] | | | | |
| 8—CO | – | 171.0 | – | |
| 9—$CH_\alpha$ | 4.26, 1H, q, 8.0 | 50.4 | 8, 10 | 10 |
| 10—$CH_\beta$ | 1.49, 2H, m | 38.8 | 9, 11, 13 | 9 |
| 11—$CH_\gamma$ | 1.53, 1H, m | 24.2 | | 10 |
| 12—$CH_{\delta,1}$ | 0.87, 3H, d, 6.5 | 22.9 | 10, 11, 13 | 11 |
| 13—$CH_{\delta,2}$ | 0.80, 3H, d, 6.5 | 21.4 | 10, 11, 12 | 11 |
| 14—NH | 8.12, 1H, d, 8.5 | – | (9), 15 | 9 |

**Table S1.** *Cont.*

| | | | | |
|---|---|---|---|---|
| L-*allo-Thr*[1] | | | | |
| 15—CO | − | 170.7 | − | |
| 16—$CH_\alpha$ | 3.86, 1H, m | 59.9 | 15, 17 | 17 |
| 17—$CH_\beta$ | 3.88, 1H, m | 65.1 | (16) | 16, 18 |
| 17—OH | 4.70, 1H, d, 4.6 | − | 16, 17, 18 | 17 |
| 18—$CH_\gamma$ | 1.03, 3H, d, 6.0 | 20.0 | 16, 17 | 17 |
| 19—NH | 8.09, 1H, d, 6.0 | − | (16), 20 | 16 |
| *D-Ser* | | | | |
| 20—CO | − | 170.0 | − | |
| 21—$CH_\alpha$ | 4.38, 1H, m | 54.5 | 20, 22, 24 | 22 |
| 22—$CH_\beta$ | 3.54, 2H, m | 61.6 | 20, (21) | 21 |
| 22—OH | 4.86, 1H, t, 6.0 | − | (21), 22 | 22 |
| 23—NH | 7.53, 1H, d, 8.0 | − | 24 | 21 |
| L-*allo-Thr*[2] | | | | |
| 24—CO | − | 168.4 | − | |
| 25—$CH_\alpha$ | 4.40, 1H, d, 9.0 | 56.2 | 24, 29 | |
| 26—$CH_\beta$ | 5.35, 1H, qd, 6.0, <1 | 69.6 | 1 | 27 |
| 27—$CH_\gamma$ | 1.10, 3H, d, 6.5 | 17.0 | 25, 26 | 26 |
| 28—NH | 8.40, 1H, d, 9.5 | | 29 | 25 |
| *Leu*[3] | | | | |
| 29—CO | − | 173.1 | − | |
| 30—$CH_\alpha$ | 4.37, 1H, m | 51.7 | 29, 31 | 31 |
| 31—$CH_\beta$ | 1.48, 2H, m | 40.3 | 29, 30, 32 | |
| 32—$CH_\gamma$ | 1.62, 1H, m | 24.2 | 33 | |
| 33—$CH_{\delta,1}$ | 0.92, 3H, d, 6.5 | 22.6 | 31, 32, 34 | 32 |
| 34—$CH_{\delta,2}$ | 0.87, 3H, d, 6.5 | 21.9 | 31, 32, 33 | 32 |
| 35—NH | 8.20, 1H, d, 6.5 | − | 30, 31, 36 | 30 |
| *Fatty acid* | | | | |
| 36—CO | − | 171.8 | − | |
| 37 | ~2.24, 2H, m | 43.3 | 36, 38, (39) | 38 |
| 38 | 3.78, 1H, m | 67.4 | | 37 |
| 38—OH | 4.62, 1H, d, 5.0 | − | 37, 38, 39 | 38 |
| 39 | 1.34, 1H, m | 36.2 | (38), 40 | (38), 40 |
| | 1.30, 1H, m | | | |
| 40 | 1.42, 1H, m | 25.1 | 41, 42 | 41 |
| | 1.28, 1H, m | | | |
| 41 | 1.96, 2H, m | 26.6 | 43 | (40), 42 |
| 42 | 5.31, 1H, m | 129.6 | 44 | 41, 43 |
| 43 | 5.31, 1H, m | 129.6 | 43 | 42, 44 |
| 44 | 1.96, 2H, m | 26.5 | 42 | 43, 45 |
| 45 | 1.28, 2H, m | 29.0 | | 44 |
| 46 | 1.24, 2H, m | 28.3 | | 47 |
| 47 | 1.22, 2H, m | 31.1 | 45 | 46 |
| 48 | 1.25, 2H, m | 22.1 | 47, 49 | 47, 49 |
| 49 | 0.84, 3H, t, 7.0 | 13.9 | 47, 48 | 48 |

**Table S2.** NMR table for Ngercheumicin G.

| Position, Type | $\delta_H$ (ppm), #H, Multiplicity, *J* (Hz) | $\delta_C$ (ppm) | HMBC Connectivity | H2BC Connectivity |
|---|---|---|---|---|
| *Leu[1]* | | | | |
| 1—CO | − | 170.8 | − | |
| 2—$CH_\alpha$ | 4.37, 1H, m | 51.1 | 1, 3, 4, 8 | 3 |
| 3—$CH_\beta$ | 1.61, 1H, m | 40.4 | 1, 2, 4, 5, 6 | 2, 4 |
| | 1.55, 1H, m | | | |
| 4—$CH_\gamma$ | 1.49, 1H, m | 24.5 | 3, 5, 6 | 3 |
| 5—$CH_{\delta,1}$ | 0.88, 3H, d, 6.5 | 22.3 | 3, 4, 6 | 4 |
| 6—$CH_{\delta,2}$ | 0.85, 3H, d, 6.5 | 22.5 | 3, 4, 5 | 4 |
| 7—NH | 7.73, 1H, d, 6.5 | − | (1), 2, 3, 8 | 2 |
| *Leu[2]* | | | | |
| 8—CO | − | 171.0 | − | |
| 9—$CH_\alpha$ | 4.26, 1H, q, 8.0 | 50.4 | 8, 10, 11 | 10 |
| 10—$CH_\beta$ | 1.49, 2H, m | 38.8 | 9, 11, 12, 13 | 9, 11 |
| 11—$CH_\gamma$ | 1.53, 1H, m | 24.2 | (9), 10, 12, 13 | 10 |
| 12—$CH_{\delta,1}$ | 0.87, 3H, d, 6.5 | 22.9 | 10, 11, 13 | 11 |
| 13—$CH_{\delta,2}$ | 0.80, 3H, d, 6.5 | 21.4 | 10, 11, 12 | 11 |
| 14—NH | 8.11, 1H, d, 8.5 | − | 9, (10), 15 | 9 |
| L-*allo-Thr[1]* | | | | |
| 15—CO | − | 170.7 | − | |
| 16—$CH_\alpha$ | 3.86, 1H, m | 59.9 | 15, 17, (18), 20 | 17 |
| 17—$CH_\beta$ | 3.88, 1H, m | 65.1 | 16 | 16, 18 |
| 17—OH | 4.69, 1H, d, 4.6 | − | 16, 17, 18 | 17 |
| 18—$CH_\gamma$ | 1.03, 3H, d, 6.0 | 20.1 | 16, 17 | 17 |
| 19—NH | 8.09, 1H, d, 6.0 | − | 16, 17, 20 | 16 |
| *D-Ser* | | | | |
| 20—CO | − | 170.0 | − | |
| 21—$CH_\alpha$ | 4.38, 1H, m | 54.6 | 20, 22, (24) | 22 |
| 22—$CH_\beta$ | 3.54, 2H, m | 61.5 | 20, 21 | 21 |
| 22—OH | 4.86, 1H, t, 6.0 | − | 21, 22 | 22 |
| 23—NH | 7.53, 1H, d, 8.0 | − | (20), 21, 22, 24 | 21 |
| L-*allo-Thr[2]* | | | | |
| 24—CO | − | 168.4 | − | |
| 25—$CH_\alpha$ | 4.39, 1H, d, 9.0 | 56.2 | 24, 26, 27, 29 | |
| 26—$CH_\beta$ | 5.34, 1H, qd, 6.0, <1 | 69.6 | 1, 24, 27 | 27 |
| 27—$CH_\gamma$ | 1.10, 3H, d, 6.5 | 17.0 | 25, 26 | 26 |
| 28—NH | 8.39, 1H, d, 9.5 | | 25, (26), 29 | 25 |
| *Leu[3]* | | | | |
| 29—CO | − | 173.2 | − | |
| 30—$CH_\alpha$ | 4.37, 1H, m | 51.8 | 29, 31, 32, 36 | 31 |
| 31—$CH_\beta$ | 1.48, 2H, m | 40.2 | 29, 30, 32, 33 | 30 |
| 32—$CH_\gamma$ | 1.62, 1H, m | 24.2 | 31, 33 | 31, 33, 34 |
| 33—$CH_{\delta,1}$ | 0.92, 3H, d, 6.5 | 22.6 | 31, 32, 34 | 32 |
| 34—$CH_{\delta,2}$ | 0.87, 3H, d, 6.5 | 21.9 | 31, 32, 33 | 32 |
| 35—NH | 8.20, 1H, d, 6.5 | − | (29), 30, 31, 36 | 30 |
| *Fatty acid* | | | | |

**Table S2.** *Cont.*

| | | | | |
|---|---|---|---|---|
| 36—CO | − | 171.9 | − | |
| 37 | 2.24, 1H, dd, 14.0, 6.0 | 43.4 | 36, 38, 39 | 38 |
| | 2.22, dd, 14.0, 7.0 | | | |
| 38 | 3.77, 1H, m | 67.5 | 36, 40 | 37, 39 |
| 38—OH | 4.60, 1H, d, 5.0 | − | 37, 38, 39 | 38 |
| 39 | 1.31, 2H, m | 36.6 | 37, 38, 41 | 38, 40 |
| 40 | 1.34, 1H, m | 24.8 | | |
| | 1.22, 1H, m | | | |
| 41 | 1.23, 2H, m | ~29 | | |
| 42 | ~1.2, 2H, m | ~29 | | |
| 43 | ~1.2, 2H, m | ~29 | | |
| 44 | ~1.2, 2H, m | ~29 | | |
| 45 | ~1.2, 2H, m | ~29 | | |
| 46 | 1.21, 2H, m | 29.1 | | |
| 47 | 1.22, 2H, m | 31.3 | | 46, 48 |
| 48 | 1.25, 2H, m | 22.1 | 47, 49 | 47, 49 |
| 49 | 0.84, 3H, t, 7.0 | 13.9 | 47, 48 | 48 |

**Table S3.** NMR table for ngercheumicin H.

| Position, Type | $\delta_H$ (ppm), #H, Multiplicity, *J* (Hz) | $\delta_C$ (ppm) | HMBC Connectivity | H2BC Connectivity |
|---|---|---|---|---|
| *Leu*[1] | | | | |
| 1—CO | − | 170.8 | − | |
| 2—$CH_\alpha$ | 4.37, 1H, m | 51.1 | 1, 3, 4, 8 | |
| 3—$CH_\beta$ | 1.62, 1H, m | 40.4 | 1, 2, 4, 5, 6 | |
| | 1.56, 1H, m | | | |
| 4—$CH_\gamma$ | 1.50, 1H, m | 24.5 | 2, 3, 5, 6 | |
| 5—$CH_{\delta,1}$ | 0.89, 3H, d, 6.5 | 22.3 | 3, 4, 6 | 4 |
| 6—$CH_{\delta,2}$ | 0.85, 3H, d, 6.5 | 22.5 | 3, 4, 5 | 4 |
| 7—NH | 7.73, 1H, d, 6.5 | − | (1), 2, 3, 8 | 2 |
| *Leu*[2] | | | | |
| 8—CO | − | 171.0 | − | |
| 9—$CH_\alpha$ | 4.25, 1H, q, 8.0 | 50.4 | 8, 10, 11 | 10 |
| 10—$CH_\beta$ | 1.49, 2H, m | 38.8 | 9, 11, 13 | 9, 11 |
| 11—$CH_\gamma$ | 1.54, 1H, m | 24.1 | 9, 10, 13 | 10 |
| 12—$CH_{\delta,1}$ | 0.87, 3H, d, 6.5 | 22.9 | 10, 11, 13 | 11 |
| 13—$CH_{\delta,2}$ | 0.80, 3H, d, 6.5 | 21.4 | 10, 11, 12 | 11 |
| 14—NH | 8.11, 1H, d, 8.5 | − | 9, 10, 15 | 9 |
| L-*allo-Thr*[1] | | | | |
| 15—CO | − | 170.7 | − | |
| 16—$CH_\alpha$ | 3.86, 1H, m | 59.9 | 15, 17, 18, 20 | 17 |
| 17—$CH_\beta$ | 3.88, 1H, m | 65.1 | 16 | 16, 18 |
| 17—OH | 4.69, 1H, d, 4.6 | − | 16, 17, 18 | 17 |
| 18—$CH_\gamma$ | 1.03, 3H, d, 6.0 | 20.1 | 16, 17 | 17 |
| 19—NH | 8.09, 1H, d, 6.0 | − | 16, 17, 20 | 16 |
| D-*Ser* | | | | |

**Table S3.** *Cont.*

| | | | | |
|---|---|---|---|---|
| 20—CO | − | 170.0 | − | |
| 21—$CH_α$ | 4.38, 1H, m | 54.5 | 20, 22, 24 | 22 |
| 22—$CH_β$ | 3.54, 2H, m | 61.5 | 20, 21 | 21 |
| 22—OH | 4.86, 1H, t, 6.0 | − | 21, 22 | 22 |
| 23—NH | 7.53, 1H, d, 8.0 | − | (20), 21, 22, 24 | 21 |
| L-*allo-Thr*[2] | | | | |
| 24—CO | − | 168.4 | − | |
| 25—$CH_α$ | 4.39, 1H, d, 9.0 | 56.2 | 24, 26, 27, 29 | |
| 26—$CH_β$ | 5.34, 1H, qd, 6.0, <1 | 69.6 | 1, 24, 27 | 27 |
| 27—$CH_γ$ | 1.10, 3H, d, 6.5 | 16.9 | 25, 26 | 26 |
| 28—NH | 8.39, 1H, d, 9.5 | | 25, 26, 29 | 25 |
| *Leu*[3] | | | | |
| 29—CO | − | 173.1 | − | |
| 30—$CH_α$ | 4.37, 1H, m | 51.8 | 29, 31, 32, 36 | 31 |
| 31—$CH_β$ | 1.48, 2H, m | 40.2 | 29, 30, 32, 33 | 30 |
| 32—$CH_γ$ | 1.62, 1H, m | 24.2 | 30, 31, 34 | 31 |
| 33—$CH_{δ,1}$ | 0.91, 3H, d, 6.5 | 22.6 | 31, 32, 34 | 32 |
| 34—$CH_{δ,2}$ | 0.87, 3H, d, 6.5 | 21.9 | 31, 32, 33 | 32 |
| 35—NH | 8.20, 1H, d, 6.5 | − | (29), 30, 31, 36 | 30 |
| *Fatty acid* | | | | |
| 36—CO | − | 171.9 | − | |
| 37 | 2.25, 1H, dd, 14.0, 6.0 | 43.4 | 36, 38, 39 | 38 |
| | 2.22, 1H, dd, 14.0, 7.0 | | | |
| 38 | 3.78, 1H, m | 67.5 | 36, 40 | 37, 39 |
| 38—OH | 4.60, 1H, d, 5.0 | − | 37, 38, 39 | 38 |
| 39 | 1.32, 2H, m | 36.6 | 38, 41 | 38, 40 |
| 40 | 1.36, 1H, m | 24.8 | | 39 |
| | 1.23, 1H, m | | | |
| 41 | ~1.2, 2H, m | ~29 | | |
| 42 | 1.28, 2H, m | ~29 | 44/45 | 43 |
| 43 | 1.97, 2H, m | 26.6 | 41? | 42, 44 |
| 44 | 5.31, 1H, m | 129.6 | 43/46 | 43, 45 |
| 45 | 5.31, 1H, m | 129.6 | 43/46 | 44, 46 |
| 46 | 1.97, 2H, m | 26.6 | 48 | 45, 47 |
| 47 | 1.28, 2H, m | ~29 | 44/45 | 46 |
| 48 | 1.25, 2H, m | 28.2 | | |
| 49 | 1.22, 2H, m | 31.1 | 47, (51) | 48, 50 |
| 50 | 1.25, 2H, m | 22.0 | 48, 49, 51 | 49, 51 |
| 51 | 0.84, 3H, t, 7.0 | 13.9 | 49, 50 | 50 |

**Table S4.** NMR table for ngercheumicin I.

| Position, Type | $\delta_H$ (ppm), #H, Multiplicity, *J* (Hz) | $\delta_C$ (ppm) | HMBC Connectivity | H2BC Connectivity |
|---|---|---|---|---|
| *Leu[1]* | | | | |
| 1—CO | − | 170.8 | − | |
| 2—$CH_\alpha$ | 4.37, 1H, m | 51.1 | 1, 3, 4, 8 | |
| 3—$CH_\beta$ | 1.61, 1H, m | 40.4 | 1, 2, 4, 5, 6 | |
| | 1.56, 1H, m | | | |
| 4—$CH_\gamma$ | 1.49, 1H, m | 24.5 | 2, 3, 5, 6 | |
| 5—$CH_{\delta,1}$ | 0.88, 3H, d, 6.5 | 22.3 | 3, 4, 6 | 4 |
| 6—$CH_{\delta,2}$ | 0.85, 3H, d, 6.5 | 22.5 | 3, 4, 5 | 4 |
| 7—NH | 7.73, 1H, d, 6.5 | − | (1), 2, 3, 8 | 2 |
| *Leu[2]* | | | | |
| 8—CO | − | 171.0 | − | |
| 9—$CH_\alpha$ | 4.26, 1H, q, 8.0 | 50.4 | 8, 10, 11 | 10 |
| 10—$CH_\beta$ | 1.49, 2H, m | 38.8 | 9, 11, 13 | 9, 11 |
| 11—$CH_\gamma$ | 1.54, 1H, m | 24.1 | 9, 10, 13 | 10 |
| 12—$CH_{\delta,1}$ | 0.87, 3H, d, 6.5 | 22.9 | 10, 11, 13 | 11 |
| 13—$CH_{\delta,2}$ | 0.80, 3H, d, 6.5 | 21.4 | 10, 11, 12 | 11 |
| 14—NH | 8.11, 1H, d, 8.5 | − | 9, 10, 15 | 9 |
| L-*allo-Thr[1]* | | | | |
| 15—CO | − | 170.7 | − | |
| 16—$CH_\alpha$ | 3.86, 1H, m | 59.9 | 15, 17, 18, 20 | 17 |
| 17—$CH_\beta$ | 3.87, 1H, m | 65.1 | 16 | 16, 18 |
| 17—OH | 4.69, 1H, d, 4.6 | − | 16, 17, 18 | 17 |
| 18—$CH_\gamma$ | 1.03, 3H, d, 6.0 | 20.1 | 16, 17 | 17 |
| 19—NH | 8.09, 1H, d, 6.0 | − | 16, 17, 20 | 16 |
| D-*Ser* | | | | |
| 20—CO | − | 170.0 | − | |
| 21—$CH_\alpha$ | 4.38, 1H, m | 54.5 | 20, 22, 24 | 22 |
| 22—$CH_\beta$ | 3.54, 2H, m | 61.5 | 20, 21 | 21 |
| 22—OH | 4.86, 1H, t, 6.0 | − | 21, 22 | 22 |
| 23—NH | 7.53, 1H, d, 8.0 | − | (20), 21, 22, 24 | 21 |
| L-*allo-Thr[2]* | | | | |
| 24—CO | − | 168.4 | − | |
| 25—$CH_\alpha$ | 4.39, 1H, d, 9.0 | 56.2 | 24, 26, 27, 29 | |
| 26—$CH_\beta$ | 5.35, 1H, qd, 6.0, <1 | 69.6 | 1, 24, 27 | 27 |
| 27—$CH_\gamma$ | 1.10, 3H, d, 6.5 | 17.0 | 25, 26 | 26 |
| 28—NH | 8.39, 1H, d, 9.5 | | 25, 26, 29 | 25 |
| *Leu[3]* | | | | |
| 29—CO | − | 173.2 | − | |
| 30—$CH_\alpha$ | 4.37, 1H, m | 51.8 | 29, 31, 32, 36 | 31 |
| 31—$CH_\beta$ | 1.48, 2H, m | 40.2 | 29, 30, 32, 33 | 30 |
| 32—$CH_\gamma$ | 1.63, 1H, m | 24.2 | 30, 31, 34 | |
| 33—$CH_{\delta,1}$ | 0.92, 3H, d, 6.5 | 22.6 | 31, 32, 34 | 32 |

**Table S4.** *Cont.*

| | | | | |
|---|---|---|---|---|
| 34—$CH_{\delta,2}$ | 0.87, 3H, d, 6.5 | 21.9 | 31, 32, 33 | 32 |
| 35—NH | 8.20, 1H, d, 6.5 | − | (29), 30, 31, 36 | 30 |
| *Fatty acid* | | | | |
| 36—CO | − | 171.9 | − | |
| 37 | 2.25, 1H, dd, 14.0, 7.0 2.22, 1H, dd, 14.0, 6.0 | 43.4 | 36, 38, 39 | 38 |
| 38 | 3.78, 1H, m | 67.5 | 36, 40 | 37, 39 |
| 38—OH | 4.59, 1H, d, 5.0 | − | 37, 38, 39 | 38 |
| 39 | 1.32, 2H, m | 36.6 | 38, 41 | 38, 40 |
| 40 | 1.35, 1H, m<br>1.22, 1H, m | 24.8 | | |
| 41 | 1.23, 2H, m | 29.1 | | |
| 42 | ~1.2, 2H, m | ~29 | | |
| 43 | ~1.2, 2H, m | ~29 | | |
| 44 | ~1.2, 2H, m | ~29 | | |
| 45 | ~1.2, 2H, m | ~29 | | |
| 46 | ~1.2, 2H, m | ~29 | | |
| 47 | ~1.2, 2H, m | ~29 | | |
| 48 | ~1.2, 2H, m | ~29 | | |
| 49 | 1.22, 2H, m | 31.3 | | 48, 50 |
| 50 | 1.25, 2H, m | 22.1 | 48, 49, 51 | 49, 51 |
| 51 | 0.84, 3H, t, 7.0 | 13.9 | 49, 50 | 50 |
